# Ectopic expression of *ORANGE* promotes carotenoid accumulation and fruit development in tomato

**DOI:** 10.1111/pbi.12945

**Published:** 2018-05-31

**Authors:** Mohammad Yazdani, Zhaoxia Sun, Hui Yuan, Shaohua Zeng, Theodore W. Thannhauser, Julia Vrebalov, Qiyue Ma, Yimin Xu, Zhangjun Fei, Joyce Van Eck, Shiping Tian, Yaakov Tadmor, James J. Giovannoni, Li Li

**Affiliations:** ^1^ Robert W. Holley Center for Agriculture and Health USDA‐ARS Cornell University Ithaca NY USA; ^2^ Plant Breeding and Genetics Section School of Integrative Plant Science Cornell University Ithaca NY USA; ^3^ College of Agriculture Institute of Agricultural Bioengineering Shanxi Agricultural University Taigu Shanxi China; ^4^ Guangdong Provincial Key Laboratory of Applied Botany South China Botanical Garden Chinese Academy of Sciences Guangzhou China; ^5^ Boyce Thompson Institute Cornell University Ithaca NY USA; ^6^ Key Laboratory of Plant Resources Institute of Botany Chinese Academy of Sciences Beijing China; ^7^ Plant Science Institute Israeli Agricultural Research Organization Newe Yaar Research Center Ramat Yishai Israel

**Keywords:** *OR*, carotenoids, plastids, early flowering, fruit set, tomato

## Abstract

Carotenoids are critically important to plants and humans. The *ORANGE* (*OR*) gene is a key regulator for carotenoid accumulation, but its physiological roles in crops remain elusive. In this study, we generated transgenic tomato ectopically overexpressing the Arabidopsis wild‐type *OR* (*At* *OR*^*WT*^) and a ‘golden SNP’‐containing *OR* (*At* *OR*^*H*^ ^*is*^). We found that *At* *OR*^*H*^ ^*is*^ initiated chromoplast formation in very young fruit and stimulated carotenoid accumulation at all fruit developmental stages, uncoupled from other ripening activities. The elevated levels of carotenoids in the *AtOR* lines were distributed in the same subplastidial fractions as in wild‐type tomato, indicating an adaptive response of plastids to sequester the increased carotenoids. Microscopic analysis revealed that the plastid sizes were increased in both *At* *OR*^*WT*^ and *At* *OR*^*H*^ ^*is*^ lines at early fruit developmental stages. Moreover, *AtOR* overexpression promoted early flowering, fruit set and seed production. Ethylene production and the expression of ripening‐associated genes were also significantly increased in the *AtOR* transgenic fruit at ripening stages. RNA‐Seq transcriptomic profiling highlighted the primary effects of *OR* overexpression on the genes in the processes related to RNA, protein and signalling in tomato fruit. Taken together, these results expand our understanding of *OR* in mediating carotenoid accumulation in plants and suggest additional roles of *OR* in affecting plastid size as well as flower and fruit development, thus making *OR* a target gene not only for nutritional biofortification of agricultural products but also for alteration of horticultural traits.

## Introduction

Carotenoids are 40‐carbon isoprenoids that are widely distributed in plants, fungi, algae and bacteria (Fraser and Bramley, 2004; Hirschberg, 2001; Nisar *et al*., 2015; Ruiz‐Sola and Rodríguez‐Concepción, 2012; Sun *et al*., 2018; Yuan *et al*., 2015b). In plants, carotenoids act as structural components of photosynthetic apparatus, protect plants from photooxidative damage and serve as precursors for the biosynthesis of volatiles, phytohormones and additional signalling molecules. In humans, carotenoids provide provitamin A and antioxidants in reducing the onset of some chronic diseases (Fiedor and Burda, 2014; Fraser and Bramley, 2004).

Carotenoids are synthesized in nearly all types of plastids in plants and accumulate to high levels in chromoplasts of fruit, roots, tubers and flowers (Sun *et al*., 2018; Yuan *et al*., 2015b). The first committed step in the carotenoid biosynthesis pathway starts with the condensation of two molecules of geranylgeranyl diphosphate (GGPP) into phytoene by phytoene synthase (PSY). After four subsequent steps of desaturation and isomerization, phytoene is converted into lycopene, the red carotenoid characteristic of tomato. Lycopene can be further metabolized to produce α‐ and β‐carotene, and then xanthophylls. While PSY is the major rate‐limiting enzyme for carotenogenesis and directs the metabolic flux towards carotenoid biosynthesis, ORANGE (OR) is a master switch governing chromoplast biogenesis and carotenoid accumulation in orange cauliflower and melon fruit (Lu *et al*., 2006; Tzuri *et al*., 2015).

*OR* encodes a plastidial DnaJ cysteine‐rich domain‐containing protein and is highly conserved among plant species (Lu *et al*., 2006; Tzuri *et al*., 2015). Recent studies reveal that OR has dual roles in post‐transcriptionally regulating PSY and promoting chromoplast biogenesis for carotenoid accumulation in plants (Chayut *et al*., 2017; Park *et al*., 2016; Welsch *et al*., 2018; Yuan *et al*., 2015a; Zhou *et al*., 2015). Expression of a wild‐type *OR* (*OR* ^*WT*^) results in carotenoid accumulation in multiple plant species (Bai *et al*., 2016; Berman *et al*., 2017; Park *et al*., 2015; Yuan *et al*., 2015a), likely due to its role in regulating PSY activity. Investigation of melon fruit flesh colour variation identified a ‘golden SNP’ in *OR* responsible for β‐carotene accumulation in a broad germplasm collection (Tzuri *et al*., 2015). This ‘golden SNP’ replaces a highly conserved arginine with histidine in melon OR. Expression of *AtOR* ^*His*^ that mimics the melon ‘golden SNP’ greatly elevates carotenoid accumulation in Arabidopsis calli, primarily due to its unique ability in mediating chromoplast biogenesis (Yuan *et al*., 2015a). Although overexpression of *OR* has been shown to affect carotenoid accumulation in nongreen tissues (Bai *et al*., 2016; Berman *et al*., 2017; Campbell *et al*., 2015; Lopez *et al*., 2008; Yuan *et al*., 2015a), whether *OR* enhances carotenoid accumulation in carotenoid‐enriched tissues and whether it affects other physiological processes remain unknown.

Tomato (*Solanum lycopersicum*) fruit accumulate exceptional levels of carotenoids at maturity and are used as a primary model to study carotenogenesis and fruit development (Giovannoni *et al*., 2017; Liu *et al*., 2015; Seymour *et al*., 2013). Tomato fruit (carpel) expansion occurs upon successful pollination and fertilization. Both sugar metabolism and phytohormones are known to affect fruit set (Kumar *et al*., 2014; Ruan *et al*., 2012). Young tomato fruit are green containing chloroplasts. The transition from chloroplasts to chromoplasts with the loss of photosynthetic membrane integrity marks the initiation of tomato fruit ripening. During the ripening process, the green colour dissipates and the distinctive red colour with massive lycopene and some β‐carotene accumulation occurs. Constitutive overexpression of *PSY1*, the key gene for the biosynthesis of fruit carotenoids, results in altering carotenoid composition and plastid type at early stages of tomato fruit development (Fraser *et al*., 2007). The use of a heterologous *PDS* from Arabidopsis bypasses a feedback regulation and elevates carotenoids in transgenic tomato fruit (McQuinn *et al*., 2017). Characterization of transgenic lines expressing a bacterial desaturase (*CrtI*) in *tangerine* and *old gold crimson* tomato mutants provides further evidence of carotenoid pathway regulatory complexity when attempting to modulate carotenoid homeostasis (Enfissi *et al*., 2017).

In this study, we investigated the impact of *AtOR* ^*WT*^ and *AtOR* ^*His*^ overexpression on tomato fruit carotenoid accumulation and fruit development at molecular, biochemical and cytological levels. We found that ectopic expression of the *AtOR* transgenes further enhanced carotenoid levels in tomato fruit that are already enriched with carotenoids. *AtOR* ^*His*^ was able to convert chloroplasts into chromoplasts at early fruit developmental stages. Moreover, overexpression of *AtOR* enhanced plastid size and promoted early flowering, fruit set and seed production in tomato. These results demonstrate that *OR* is an important factor broadly influencing carotenoid biosynthesis and chromoplast biogenesis, and may play a role in affecting flowering and fruit development.

## Results

### Constitutive expression of *AtOR* ^*His*^ enhances carotenoid content in fruit and flowers

To investigate the effect of *OR* on carotenoid accumulation in tomato fruit, we introduced *AtOR* ^*WT*^ and *AtOR* ^*His*^ overexpression constructs into tomato cv M82. More than ten positive primary transgenic lines (T0) were generated for each construct. Increased expression of OR protein levels in some transgenic lines was confirmed by Western blot analysis of leaf samples (Figure S1). Based on the analysis, three transgenic lines of *AtOR* ^*WT*^ (5, 8 and 20) and three *AtOR* ^*His*^ (17b, 21b, and 23a) were selected for the subsequent study.

**Figure 1:**
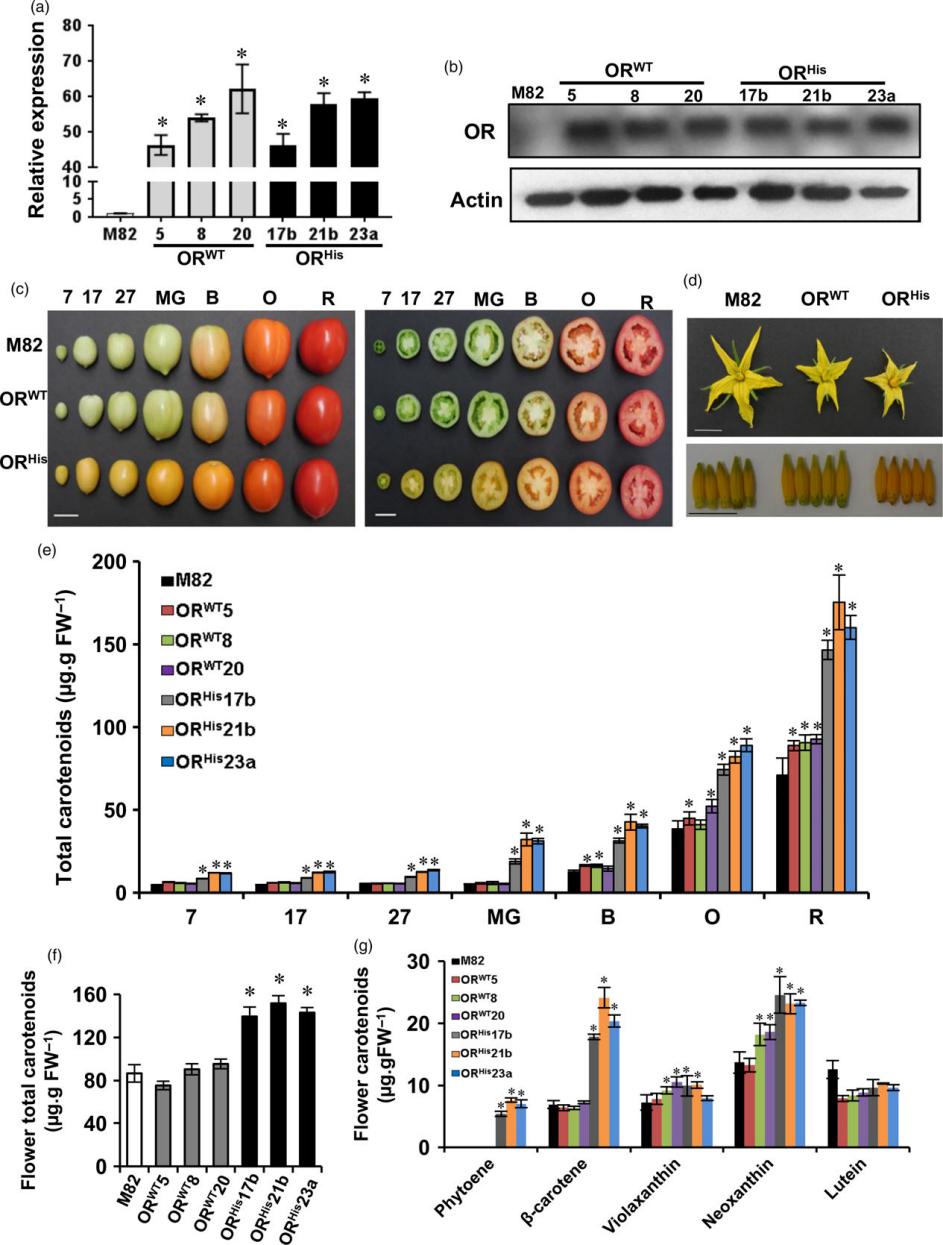
Characterization of the *AtOR* T1 transgenic lines. (a) qRT‐PCR analysis of the *OR* expression at mature green stage in the *At* *OR*^*WT*^ (5, 8 and 20) and *At* *OR*^*H*^ ^*is*^ (17b, 21b and 23a) lines. (b) Western blot analysis of OR protein levels at mature green stage. Actin shows protein loading. (c) Representative phenotype of the tomato fruit at different developmental stages of M82, *At* *OR*^*WT*^ 20 and *At* *OR*^*H*^ ^*is*^ 21b (Bar = 2 cm). (d) Phenotype of the tomato flowers and stamens of these lines (Bar = 1 cm). (e) Total carotenoid levels at different fruit developmental stages. (f) Total carotenoid levels in flowers. (g) Carotenoid profiles in flowers. Bar data are the means of three biological replicates ± SE. *P <* *0.05. B, breaker; FW, fresh weight; 7, 17 and 27 days postanthesis; MG, mature green; O, orange; R, red stage.

Expression of the *OR* gene and OR protein in mature green (MG) fruit of M82 and the *AtOR* T1 transgenic lines were examined by qRT‐PCR and Western blot analysis, respectively. Both OR transcript and protein levels were dramatically elevated in these *AtOR* ^*WT*^ and *AtOR* ^*His*^ transgenic lines in comparison with M82 (Figure 1a,b). Comparable OR transcript and protein levels were observed between the *AtOR* ^*WT*^ and *AtOR* ^*His*^ lines. High levels of OR expression were also observed in leaves of the *AtOR* ^*WT*^ and *AtOR* ^*His*^ transgenic plants (Figure S2a,b)

The *AtOR* ^*WT*^ and *AtOR* ^*His*^ transgenes have been shown to alter the colour of Arabidopsis calli differently (Yuan *et al*., 2015a). Similarly, expression of *AtOR* ^*WT*^ and *AtOR* ^*His*^ showed different effects on tomato fruit colour. While the colour at different fruit developmental stages was almost the same between M82 and *AtOR* ^*WT*^ lines, noticeable difference was observed between M82 and *AtOR* ^*His*^ lines (Figure 1c). Fruit of all three *AtOR* ^*His*^ transgenic lines showed visible orange colour from very young fruit to the orange (O) stage (Figure 1c). Similarly, while the flowers of M82 and *AtOR* ^*WT*^ transgenic lines shared similar hue, the flowers of all three *AtOR* ^*His*^ transgenic lines had stamens with intense orange colour (Figure 1d). The results indicate that *AtOR* ^*His*^ affected carotenoid accumulation in both tomato fruit and flowers.

Carotenoid levels of fruit at 7, 17, 27 days postanthesis (DPA), mature green (MG), breaker (B), orange (O) and red (R) stages (Alba *et al*., 2005) were analysed by UPC^2^. The total carotenoid levels in the three *AtOR* ^*His*^ T1 lines were significantly higher than those in M82 and *AtOR* ^*WT*^ lines at all fruit developmental stages (Figure 1e, Table 1). The largest difference was observed at the MG stage. Carotenoid contents in MG, B, O and R stages of the *AtOR* ^*His*^ lines were approximately 6.4‐, 3.5‐, 2.3‐ and 2.5‐fold higher, respectively, than those in M82. Noticeably, small but significantly increased total carotenoid accumulation was detected in the *AtOR* ^*WT*^ transgenic lines especially at the R stage in comparison with M82 control.

**Table 1:** Carotenoid profile and levels at various fruit developmental stages in M82 and the *AtOR* T1 transgenic lines

Genotype	Phytoene	Phytofluene	ζ‐carotene	Lycopene	α‐carotene	β‐carotene	Lutein	Others	Total
7 DPA									
M82	0	0	0	0	0	1.93 ± 0.02	2.37 ± 0.02	0.28 ± 0.01	4.57 ± 0.06
OR^WT^5	0	0	0	0	0	**2.66 ± 0.14**	**3.07 ± 0.05**	**0.37 ± 0.02**	6.09 ± 0.19
OR^WT^8	0	0	0	0	0	**2.80 ± 0.08**	2.37 ± 0.13	0.25 ± 0.01	5.42 ± 0.23
OR^WT^20	0	0	0	0	0	**2.34 ± 0.12**	**2.57 ± 0.06**	**0.38 ± 0.01**	5.29 ± 0.18
OR^HI,^17b	0	0.32 ± 0.11	0	0	0	**3.70 ± 0.02** a	**4.22 ± 0.13** a	0.28 ± 0.01	**8.52 ± 0.14** a
OR^His^21b	0.33 ± 0.11	0.43 ± 0.13	0	0	0	**5.24 ± 0.07** a	**5.74 ± 0.08** a	**0.33 ± 0.01**	**12.07 ± 0.10** a
OR^His^23a	0.25 ± 0.02	0.33 ± 0.16	0	0	0	**6.12 ± 0.16** a	**4.37 ± 0.08** a	**0.49 ± 0.02** a	**11.76 ± 0.26** a
17 DPA									
M82	0	0	0	0	0	1.93 ± 0.07	2.08 ± 0.10	0.28 ± 0.01	4.29 ± 0.27
OR^WT^5	0	0	0	0	0	**2.46 ± 0.06**	**2.56 ± 0.05**	0.29 ± 0.01	5.30 ± 0.10
OR^WT^8	0	0	0	0	0	**2.57 ± 0.04**	**2.71 ± 0.06**	0.28 ± 0.02	5.56 ± 0.31
OR^WT^20	0	0	0	0	0	**2.54 ± 0.02**	**2.44 ± 0.02**	0.30 ± 0.01	5.28 ± 0.22
OR^His^17b	0.23 ± 0.01	0.41 ± 0.02	0	0	0	**4.35 ± 0.02** a	**3.64 ± 0.02** a	0.25 ± 0.01	**8.89 ± 0.10** a
OR^His^21b	0.26 ± 0.05	0.47 ± 0.01	0	0	0	**6.52 ± 0.10** a	**4.64 ± 0.05** a	0.30 ± 0.02	**12.19 ± 0.20** a
OR^His^23a	0.34 ± 0.03	0.58 ± 0.01	0	0	0	**7.04 ± 0.20** a	**4.30 ± 0.22** a	0.24 ± 0.02	**12.50 ± 0.46** a
27 DPA									
M82	0	0	0	0	0	2.12 ± 0.04	2.65 ± 0.04	0.35 ± 0.01	5.12 ± 0.10
OR^WT^5	0	0	0	0	0	**2.40 ± 0.10**	**2.15 ± 0.06**	0.31 ± 0.02	4.85 ± 0.25
OR^WT^8	0	0	0	0	0	**2.48 ± 0.02**	**2.23 ± 0.03**	0.32 ± 0.02	5.23 ± 0.12
OR^WT^20	0	0	0	0	0	**2.57 ± 0.05**	**2.12 ± 0.04**	**0.13 ± 0.06**	5.03 ± 0.15
OR^His^17b	0.38 ± 0.03	0.40 ± 0.02	0	0	0	**5.76 ± 0.03** a	2.71 ± 0.06^a^	0.31 ± 0.01	**9.55 ± 0.20** a
OR^His^21b	0.31 ± 0.01	0.32 ± 0.03	0	0	0	**7.84 ± 0.06** a	**3.80 ± 0.03** a	**0.28 ± 0.01**	**12.56 ± 0.21** a
OR^His^23a	0.35 ± 0.04	0.61 ± 0.02	0	0	0	**8.89 ± 0.18** a	**3.53 ± 0.21** a	**0.25 ± 0.03**	**13.64 ± 0.37** a
MG									
M82	0	0	0	0	0	1.74 ± 0.14	2.02 ± 0.17	1.28 ± 0.06	5.05 ± 0.27
OR^WT^5	0	0	0	0	0	1.77 ± 0.29	**2.24 ± 0.34**	**1.67 ± 0.14**	5.68 ± 0.41
OR^WT^8	0	0	0	0	0	1.99 ± 0.29	**2.27 ± 0.13**	**1.58 ± 0.14**	5.84 ± 0.87
OR^WT^20	0	0	0	0	0	1.84 ± 0.28	2.08 ± 0.10	**1.43 ± 0.02**	5.37 ± 0.31
OR^His^17b	2.12 ± 0.10	2.11 ± 0.10	2.06 ± 0.30	2.33 ± 0.31	1.978 ± 0.11	**3.27 ± 0.48** a	**2.91 ± 0.13** a	**2.13 ± 0.13** a	**18.91 ± 1.53** a
OR^His^21b	4.55 ± 0.69	4.13 ± 0.53	5.13 ± 1.10	3.10 ± 0.16	2.50 ± 0.27	**4.94 ± 0.37** a	**5.15 ± 0.87** a	**2.66 ± 0.35** a	**32.18 ± 3.83** a
OR^His^23a	4.49 ± 0.15	3.91 ± 0.18	4.54 ± 0.65	3.19 ± 0.19	2.50 ± 0.13	**5.81 ± 0.44** a	**4.34 ± 0.26** a	**2.36 ± 0.11** a	**31.14 ± 1.63** a
B									
M82	1.36 ± 0.13	1.27 ± 0.14	1.28 ± 0.16	1.42 ± 0.22	0	2.59 ± 0.16	2.43 ± 0.21	2.00 ± 0.22	12.36 ± 1.24
OR^WT^5	**1.82 ± 0.02**	**1.76 ± 0.07**	**1.80 ± 0.07**	**1.97 ± 0.09**	0	**4.50 ± 0.17**	2.92 ± 0.17	**1.72 ± 0.04**	**16.49 ± 0.50**
OR^WT^8	**1.58 ± 0.15**	**1.65 ± 0.14**	**1.75 ± 0.14**	**1.94 ± 0.15**	0	**4.59 ± 0.18**	**3.08 ± 0.18**	**1.66 ± 0.12**	**16.26 ± 0.94**
OR^WT^20	1.53 ± 0.13	1.53 ± 0.18	1.61 ± 0.19	1.81 ± 0.22	0	**3.57 ± 0.37**	2.85 ± 0.33	**1.64 ± 0.10**	14.55 ± 1.50
OR^His^17b	**5.23 ± 0.17** a	**4.14 ± 0.17** a	**3.22 ± 0.10** a	**4.53 ± 0.06** a	2.39 ± 0.21	**5.38 ± 0.21** a	**4.46 ± 0.30** a	2.19 ± 0.23^a^	**31.50 ± 1.43** a
OR^His^21b	**8.09 ± 0.18** a	**6.44 ± 0.69** a	**5.65 ± 0.64** a	**4.74 ± 0.76** a	2.51 ± 0.19	**7.81 ± 0.91** a	**5.05 ± 0.62** a	2.32 ± 0.22^a^	**42.63 ± 4.74** a
OR^His^23a	**6.30 ± 0.16** a	**5.05 ± 0.14** a	**5.57 ± 0.14** a	**5.98 ± 0.17** a	2.42 ± 0.17	**8.46 ± 0.31** a	**4.27 ± 0.19** a	2.22 ± 0.14^a^	**40.28 ± 1.12** a
O									
M82	2.15 ± 0.23	1.97 ± 0.36	1.52 ± 0.03	16.18 ± 0.41	1.77 ± 0.34	7.01 ± 0.98	2.67 ± 0.04	5.33 ± 0.61	38.70 ± 4.76
OR^WT^5	**2.70 ± 0.17**	**2.20 ± 0.12**	**1.83 ± 0.06**	**21.39 ± 0.48**	1.80 ± 0.09	9.03 ± 1.96	3.43 ± 0.37	**2.49 ± 0.11**	**44.89 ± 3.95**
OR^WT^8	1.91 ± 0.06	1.91 ± 0.09	**1.69 ± 0.18**	**19.15 ± 1.12**	1.79 ± 0.11	8.76 ± 1.37	**3.45 ± 0.17**	**2.45 ± 0.17**	41.11 ± 2.82
OR^WT^20	**3.88 ± 0.18**	**3.29 ± 0.23**	**2.39 ± 0.24**	**22.44 ± 2.63**	**2.52 ± 0.19**	**9.51 ± 0.51**	**4.62 ± 0.29**	**3.67 ± 0.18**	**52.31 ± 4.05**
OR^His^17b	**6.41 ± 0.30** a	**4.87 ± 0.26** a	**4.11 ± 0.24** a	**30.58 ± 1.11** a	**3.24 ± 0.17** a	**15.77 ± 2.06** a	**5.50 ± 0.29** a	**3.86 ± 0.60**	**74.34 ± 3.27** a
OR^His^21b	**9.06 ± 0.79** a	**5.1 ± 0.84** a	**4.34 ± 0.10** a	**29.69 ± 2.14** a	**2.91 ± 0.11** a	**18.76 ± 1.12** a	**4.47 ± 0.34**	**7.64 ± 0.81** a	**81.96 ± 3.60** a
OR^His^23a	**8.95 ± 0.56** a	**6.72 ± 0.93** a	**6.28 ± 0.51** a	**32.70 ± 1.12** a	**3.21 ± 0.98** a	**20.63 ± 1.52** a	**6.73 ± 0.94** a	**3.91 ± 0.50**	**89.14 ± 3.85** a
R									
M82	5.14 ± 0.25	3.92 ± 0.14	2.46 ± 0.25	42.36 ± 4.32	2.43 ± 0.06	9.03 ± 0.72	3.29 ± 0.10	2.49 ± 0.15	71.12 ± 5.19
OR^WT^5	**5.54 ± 0.49**	**4.51 ± 0.31**	**2.74 ± 0.19**	**51.99 ± 2.19**	**3.41 ± 0.19**	**13.37 ± 1.29**	**3.87 ± 0.28**	**3.44 ± 0.40**	**88.87 ± 3.01**
OR^WT^8	**6.23 ± 0.48**	**4.24 ± 0.32**	2.64 ± 0.19	**53.63 ± 1.10**	**2.91 ± 0.38**	**14.14 ± 1.29**	3.24 ± 0.50	**3.67 ± 0.49**	**90.72 ± 4.67**
OR^WT^20	5.27 ± 0.33	**6.11 ± 1.84**	**2.96 ± 0.44**	**54.88 ± 0.84**	**3.31 ± 0.24**	**12.78 ± 0.17**	**4.55 ± 0.34**	**2.93 ± 0.10**	**92.79 ± 2.77**
OR^His^17b	**17.66 ± 0.75** a	**10.61 ± 0.51** a	**6.13 ± 1.24** a	**67.26 ± 4.26** a	**6.07 ± 0.16** a	**21.00 ± 2.84** a	**9.02 ± 1.71** a	**8.89 ± 1.03** a	**146.65 ± 5.70** a
OR^His^21b	**18.82 ± 0.91** a	**16.64 ± 1.31** a	**7.85 ± 0.59** a	**77.18 ± 7.83** a	**6.02 ± 0.22** a	**28.04 ± 1.91** a	**12.05 ± 0.48** a	**8.66 ± 0.34** a	**175.26 ± 16.52** a
OR^His^23a	**18.74 ± 2.07** a	**12.42 ± 1.57** a	**7.22 ± 0.99** a	**64.51 ± 1.17** a	**6.89 ± 0.92** a	**27.74 ± 1.58** a	**13.86 ± 1.31** a	**8.77 ± 0.52** a	**160.12 ± 7.20** a

B, breaker; DPA, days postanthesis; MG, mature green; O, orange; R, red stage.

Values (μg/g Fresh Weight) represent means ± SE from three biological replicates. Number in bold indicates a statistically significant increase in comparison with M82 control (*P *<* *0.05).

a Significant difference between *AtOR* ^*WT*^ and *AtOR* ^*His*^ lines (*P *<* *0.05).

The effect of *AtOR* transgenes on fruit carotenoid profile was analysed (Table 1). While fruit from the *AtOR* ^*WT*^ lines like M82 displayed the characteristic change from chloroplast carotenoids (β‐carotene and xanthophylls) at MG stage to chromoplast pigments (mainly lycopene) at R stage, *AtOR* ^*His*^ transgene altered the carotenoid profile at early fruit developmental stages (Table 1). The greatly increased β‐carotene levels likely contributed to the observed orange colour at early fruit developmental stages in the *AtOR* ^*His*^ lines (Figure 1c). A number of carotenoids unique to the *AtOR* ^*His*^ lines (i.e. phytoene, phytofluence, ζ‐carotene, lycopene and α‐carotene) were also detected relatively abundant at MG stage (Table 1). At the breaker and subsequent ripening stages that are characterized by the dramatic increase in carotenoid components, all the lines shared nearly similar carotenoid profiles. Lycopene was the predominant carotenoid in the fruit at R stage for all genotypes. The *AtOR* ^*His*^ transgene significantly enhanced the levels of all carotenoids at R stage, particularly the β‐carotene content, which showed 3.1‐fold increase in *AtOR* ^*His*^ 21b over M82 fruit (Table 1).

Carotenoid content and composition in flowers were also analysed. While no significant difference in total carotenoid level was detected between M82 and the *OR* ^*WT*^ lines, approximately two‐fold more carotenoids were observed in the flowers of *AtOR* ^*His*^ lines than M82 (Figure 1f). Analysis of individual carotenoids in the flowers revealed that the increased total content in the *AtOR* ^*His*^ lines was primarily due to substantially enhanced β‐carotene along with phytoene and neoxanthin (Figure 1g).

Carotenoid levels in leaf tissues of these lines were also analysed. Small (10%–26%), but significant increase in the total carotenoid levels was observed in the *AtOR* ^*His*^ lines in comparison with M82. Among *AtOR* ^*WT*^ lines, only line 8 displayed significantly higher total carotenoid level than M82 (Figure S2c).

### *AtOR* affects chromoplast development and fruit chloroplast size

OR mutant alleles have been shown to trigger the differentiation of noncoloured plastids into chromoplasts (Lopez *et al*., 2008; Lu *et al*., 2006; Yuan *et al*., 2015a). To investigate whether *AtOR* ^*His*^ also prompted the conversion of chloroplasts into chromoplasts and affected plastid development in a green tissue, we examined and compared the plastids in fruit of M82 and the *AtOR* T1 transgenic lines at 7, 17 and 27 DPA fruit developmental stages, the stages containing only chloroplasts in the wild‐type fruit.

**Figure 2:**
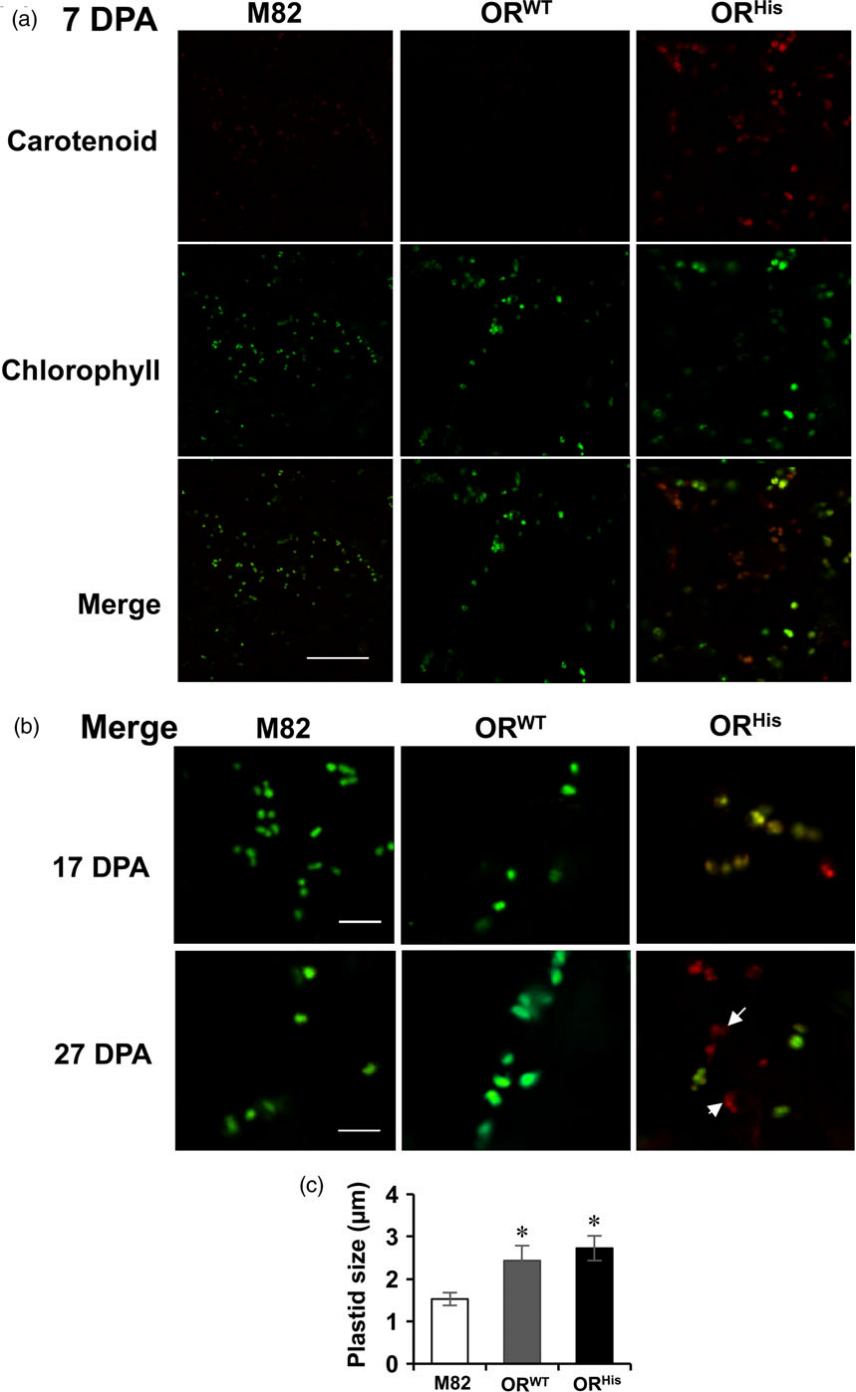
Effects of *AtOR* on plastid development and size at early fruit developmental stages of M82, *At* *OR*^*WT*^ 20 and *At* *OR*^*H*^ ^*is*^ 23a T1 transgenic lines. (a) Autofluorescence of carotenoids and chlorophylls (set as red and green signal, respectively) in 7 days postanthesis (DPA) fruit were detected using confocal microscope. Chlorophyll fluorescence was taken at 650–700 nm, and carotenoid fluorescence was taken at 500–550 nm. (b) Merged autofluorescence signals in 17 and 27 DPA fruit. The sizes of the bars in A and B are 40 and 20 μm, respectively. Arrow points to chromoplast with crystal structure. (c) Plastid sizes in 7 DPA fruit of M82, *At* *OR*^*WT*^ 20 and *At* *OR*^*H*^ ^*is*^ 23a T1 transgenic plants. Data are means of five biological replicates ± SE. *P <* *0.05.

As shown in Figure 2, the cells from M82 and *AtOR* ^*WT*^ fruit contained chloroplasts that emitted green fluorescence due to the presence of chlorophylls. However, a heterogeneous population of plastids was found in the cells from fruit of the *AtOR* ^*His*^ lines. The *AtOR* ^*His*^ fruit samples at 7 DPA contained chloroplasts that were green with chlorophylls, intermediate plastids that gave yellowish colour due to the presence of both chlorophylls and carotenoids, and fully developed chromoplasts that emitted red fluorescence (Figure 2a). At 27 DPA, chromoplasts with crystal structures in some were observed (Figure 2b). Clearly, *AtOR* ^*His*^ promoted plastid differentiation from chloroplasts at early fruit developmental stages.

Noticeably, OR appeared to enhance fruit plastid sizes. Both *AtOR* ^*WT*^ and *AtOR* ^*His*^ lines showed larger chloroplasts than M82 at early fruit developmental stages (Figure 2). However, the chromoplast sizes at late fruit ripening stages were not substantially different. Measurement of the plastid sizes in the cells of 7 DPA fruit revealed that the plastids were approximately 60% and 80% larger for *AtOR* ^*WT*^ and *AtOR* ^*His*^ lines, respectively, than M82 controls (Figure 2c). These results suggest a new role of *OR* in mediating chloroplast size.

### Sequestration of carotenoids in chromoplast subplastidial compartments is not altered in the *AtOR* fruit

**Figure 3:**
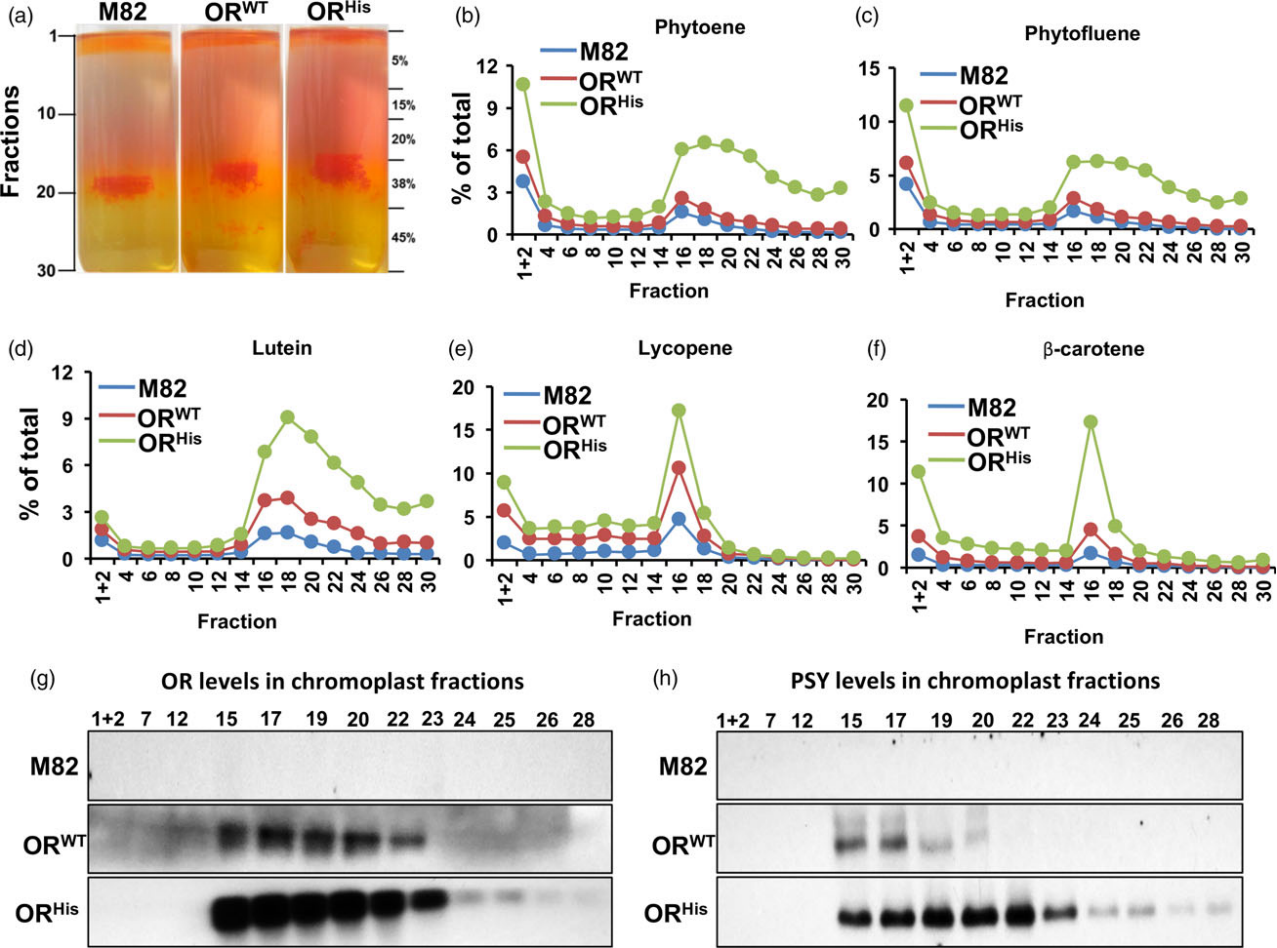
Sequestration of carotenoids in subplastidal fractions. (a) Sucrose gradient fractionation of the subchromoplast components from B + 3–5 days fruit of M82, *At* *OR*^*WT*^ 20 and *At* *OR*^*H*^ ^*is*^ 21b T1 transgenic lines. (b–f) Individual carotenoid profiles in chromoplast fractions. (g,h) Western blot analysis of OR (g) and PSY (h) proteins in subplastidal fractions. A total of 25 μg proteins for each fraction were used.

To examine whether the subplastidial localization of carotenoids in the *AtOR* fruit was altered, chromoplasts from fruit of M82, *AtOR* ^*WT*^ 20 and *AtOR* ^*His*^ 21b T1 transgenic lines at breaker +3 to 5 days stage were isolated and fractionated into three distinctive coloured sections (Figure 3a). The first section at the top of the gradient with fractions 1 and 2 is known to be plastoglobulin (PG) fraction (Nogueira *et al*., 2013). The second section between fractions 15 and 22 was observable orange with the presence of red crystal‐like structures. The third section was at the bottom of the fraction 22, which showed yellow colour and was the stroma compartment (Nogueira *et al*., 2013).

A total of 30 1‐mL fractions were collected and individual carotenoid levels in every other subplastidial fractions were analysed by UPC^2^ (Figure 3b–f). Two carotenoid intensity peaks were observed. The first peak was in fractions 1 + 2, which contained relative high concentrations of phytoene and phytofluene (Figure 3b,c). The red/orange hue in this section of the *AtOR* lines was likely due to increased levels of lycopene and β‐carotene (Figure 3e,f). The second peak was around fraction 16 with lycopene and β‐carotene that formed sharp peaks (Figure 3e,f). This section contained crystal‐like structures (Figure 3a) and belonged to submembrane compartment (Nogueira *et al*., 2013). While phytoene, phytofluene and lutein also accumulated in the second peak, they distributed throughout the later subplastidial fractions (Figure 3b–d).

The individual carotenoid intensities in each fraction were greater in the *AtOR* plants than M82. Much high levels of carotenoids, particularly β‐carotene, were observed in the *AtOR* ^*His*^ lines. However, similar carotenoid distribution profiles in the subplastidial fractions were noticed, indicating that the evaluated carotenoid accumulation in the *AtOR* lines was sequestered in the same subplastidial localizations as in M82.

Immunoblotting experiments were performed to find the subplastidial localizations of OR along with PSY, whose protein abundance is post‐translationally regulated by OR (Chayut *et al*., 2017; Zhou *et al*., 2015). While the endogenous OR and PSY protein levels were undetectable in the M82 fractions, high levels of AtOR and PSY were mainly located in the submembrane fractions 15‐23 (Figure 3g,h).

### Both *AtOR* ^*His*^ and *AtOR* ^*WT*^ promote early flowering, fruit set and seed production

**Figure 4:**
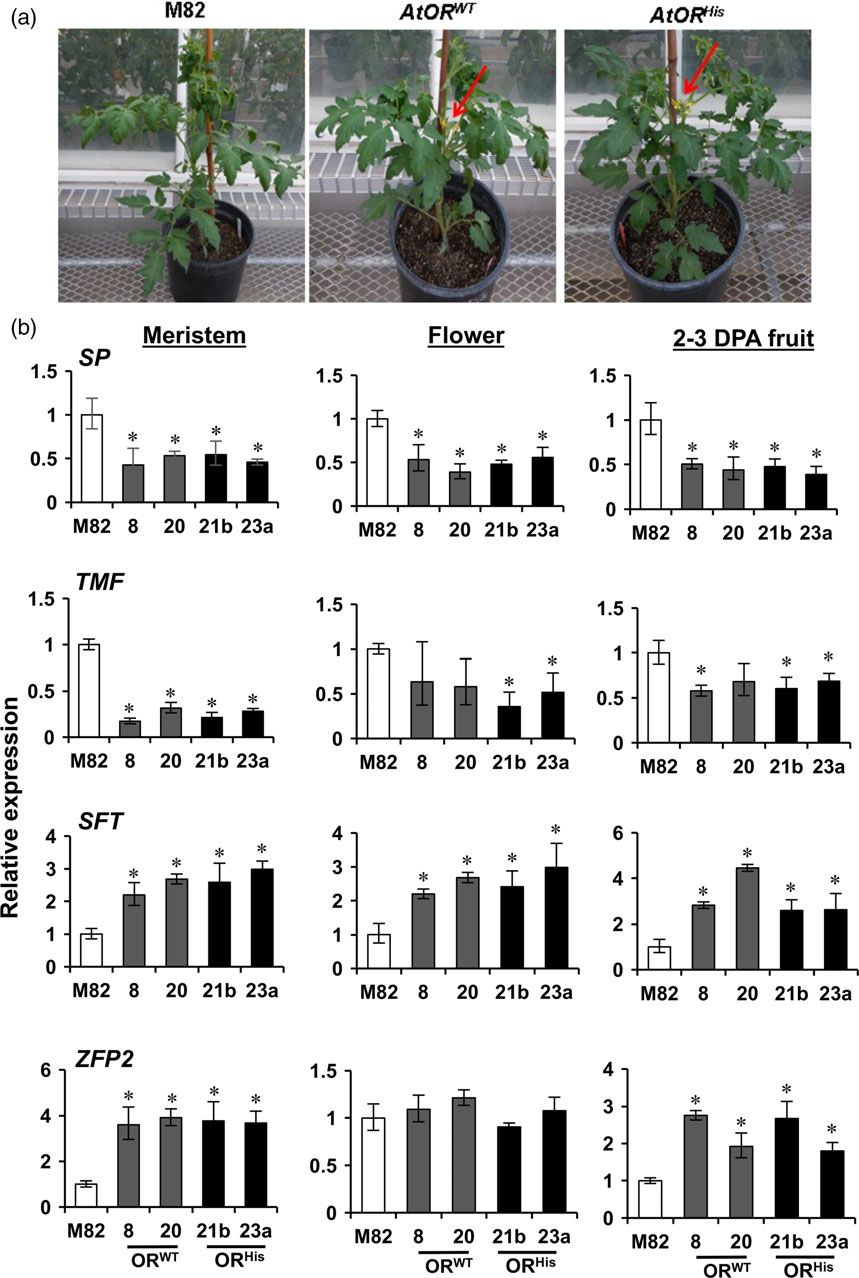
*AtOR* overexpression promotes early flowering. (a) Representative phenotype of early flowering in M82 and *AtOR* T1 transgenic plants. The arrows point flowers in 6‐week‐old *AtOR* lines but absence in M82 control. (b) *AtOR* overexpression significantly alters the expression of genes pertaining to flowering in meristems, flowers and 2–3 DPA fruit of *At* *OR*^*WT*^ (8 and 20) and *At* *OR*^*H*^ ^*is*^ (21b and 23a) lines. *SP, SELF‐PRUNING*;*TMF, TERMINATING FLOWER; SFT, SINGLE FLOWER TRUSS; ZEP2,* zinc finger transcription factor *SlZFP2*. Data are the means of three biological replicates ± SD. *P <* *0.05.

The *AtOR* T1 transgenic plants were noticed to flower nearly 7–10 days earlier than M82 plants (Figure 4a). To gain an understanding how *AtOR* transgenes promoted early flowering, the expression of a number of genes known to be involved in tomato flowering in shoot meristem, flower and young fruit at 2–3 DPA stage was tested by qRT‐PCR. These genes included two negative regulators *SELF‐PRUNING* (*SP*) and *TERMINATING FLOWER* (*TMF*), and two positive regulators *SINGLE FLOWER TRUSS* (*SFT*) and the zinc finger transcription factor *SlZFP2* (Lifschitz and Eshed, 2006; MacAlister *et al*., 2012; Pnueli *et al*., 1998; Weng *et al*., 2016). As shown in Figure 4b, with the exception for *TMF* in flowers of *AtOR* ^*WT*^ (8 and 20), and in 2–3 DPA fruit of *AtOR* ^*WT*^ 20, the transcript levels of *SP* and *TMF* were significantly down‐regulated in the *AtOR* overexpression lines. The expression levels of *SFT* and *SlZFP2* were significantly higher in meristems and 2–3 DPA fruit of the transgenic lines than M82 (Figure 4b). These results indicate that the *OR* transgenes might mediate flowering in tomato via affecting some early flowering gene expression.

**Figure 5:**
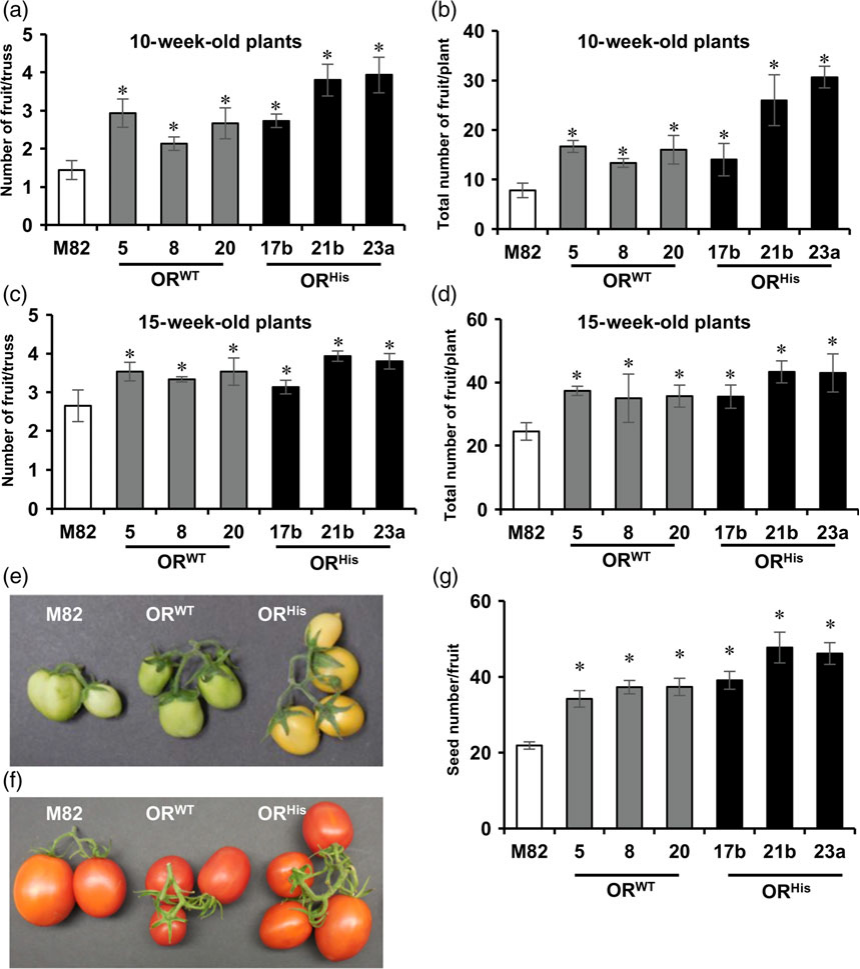
*AtOR* overexpression enhances fruit set and increases seed number in the *At* *OR*^*WT*^ (5, 8 and 20) and *At* *OR*^*H*^ ^*is*^ (17b, 21b and 23a) T1 transgenic lines. (a,c) Average number of tomato fruit per truss in 10‐ and 15‐week‐old plants, respectively. (b,d) Total number of fruit for each line in 10‐ and 15‐week‐old plants, respectively. Three plants for each line and 5–8 trusses per plant were used to count the number of fruit per truss and total fruit number. (e,f) Representative images of tomato trusses in 10‐ and 15‐week‐old plants, respectively. (g) Total seed number in red fruit. Data are the means of five fruit ± SE. *P <* *0.05.

The *AtOR* transgenic lines also produced many more but slightly smaller mature fruit than M82. To evaluate the impact of *AtOR* on fruit set, the number of fruit in each truss and the total number of the fruit for each line were counted in 10‐ and 15‐week‐old plants. Although each truss in all genotypes had a number of unopened flowers, the average fruit numbers in each truss of 10‐week‐old plants showed up to 2‐ and 2.7‐fold increase in the *AtOR* ^*WT*^ and *AtOR* ^*His*^ lines, respectively, than M82 control (Figure 5a). The total fruit numbers were significant increase with up to 2.1‐ and 3.9‐fold more fruit in the *AtOR* ^*WT*^ and *AtOR* ^*His*^ lines, respectively, than M82 (Figure 5b). Similarly, the average numbers of fruit in each truss and the total fruit numbers in 15‐week‐old plants were also significantly elevated in the *AtOR* ^*WT*^ and *AtOR* ^*His*^ lines (Figure 5c,d). The typical fruit sets for each genotype in 10‐ and 15‐week‐old plants are shown in Figure 5e,f, respectively.

Interestingly, the *AtOR* transgenic lines also produced fruit with more seeds than M82. Counting the seed numbers in ripe fruit revealed that *AtOR* ^*WT*^ and *AtOR* ^*His*^ caused an up to 1.7‐ and 2.2‐fold increase in the seed number, respectively, in comparison with M82 (Figure 5g). These results suggest novel roles of *OR* in affecting fruit development in plants.

### *OR* elevates soluble sugar content and alters relevant gene expression

**Figure 6:**
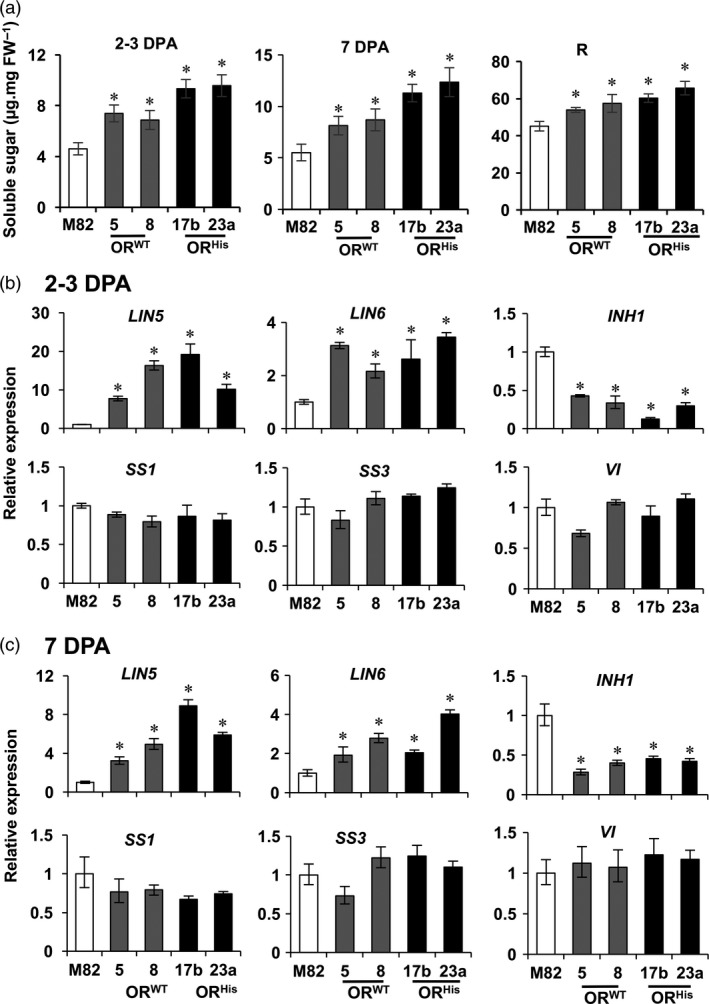
*AtOR* overexpression alters total soluble sugar content and the related gene expression. (a) Soluble sugar levels of tomato fruit at 2–3 DPA, 7 DPA and red (R) stages of M82, *At* *OR*^*WT*^ (5 and 8) and *At* *OR*^*H*^ ^*is*^ (17b and 23a) T1 transgenic lines. (b,c) Expression of genes related to sugar metabolism at 2–3 DPA and 7 DPA fruit developmental stages. *LIN5* and *LIN6, cell wall invertase 5* and *6*;*INH1, invertase inhibitor 1*,*SS1* and *SS3, sucrose synthase 1* and *3*;*VI*,* vacuolar invertase*. Data are the means of three biological replicates ± SD. *P <* *0.05.

Sugars are known to mediate fruit set and seed production (Ruan *et al*., 2012). To see whether the *OR*‐promoted fruit set and seed production were associated with altered sugar levels, we analysed total soluble sugar content in 2–3 DPA fruitlets, a time period used to examine fruit set in tomato (Palmer *et al*., 2015). Significant higher levels of sugar content were observed in both *AtOR* ^*WT*^ and *AtOR* ^*His*^ T1 transgenic lines than M82 (Figure 6a). In addition, we examined sugar levels at 7 DPA and R stages. A significant elevation also occurred at 7 DPA and continuously at R stage in these *AtOR* lines (Figure 6a), showing a capacity of *OR* in promoting the production of soluble sugars.

A cell wall invertase (LIN5) that hydrolyses sucrose into glucose and fructose has been shown to play a major role in fruit and seed set in tomato (Palmer *et al*., 2015; Zanor *et al*., 2009). To see if the *AtOR* transgenes altered the expression of genes involved in free sugar metabolism, the transcript levels of cell wall invertases *LIN5* and *LIN6*, invertase inhibitor *INH1*, sucrose synthase *SS1* and *SS3*, and vacuolar invertase *VI* in fruitlets at 2–3 DPA and 7 DPA stages were examined by qRT‐PCR. As shown in Figure 6b,c, the mRNA levels of *LIN5* and *LIN6* were significantly higher in the 2–3 DPA and 7 DPA fruit of both *AtOR* lines than M82. Invertase inhibitors suppress invertase activities (Qin *et al*., 2016). Significant reduced expression of *INH1* was observed in the *AtOR* lines, which might further elevate invertase activities. In contrast, the transcript levels of *SS1, SS3* and *VI* in the 2–3 DPA and 7 DPA fruit were not significantly affected by the *AtOR* overexpression (Figure 6b,c). The results suggest that the *OR*‐promoted fruit set and seed production might be associated with its ability to regulate free sugar levels and affect invertase and invertase inhibitor gene expression.

### *OR* enhances ethylene production and affects ripening‐related gene expression

**Figure 7:**
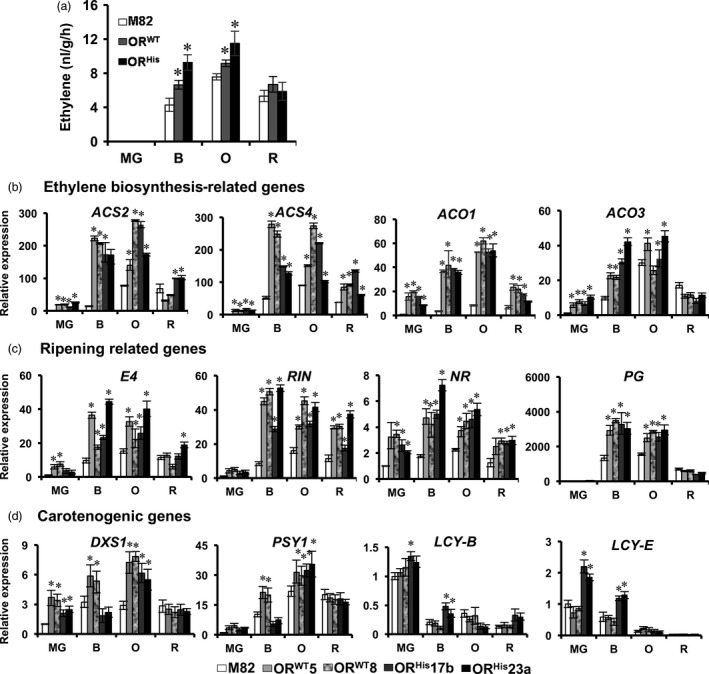
*AtOR* affects ethylene production and ripening‐related gene expression. (a) Ethylene production was enhanced at B and O stages of *At* *OR*^*WT*^ 20 and *At* *OR*^*H*^ ^*is*^ 21b T1 transgenic tomato fruit. Ethylene production was measured with 6–10 fruit for each ripening stage. (b–d) Expression of genes related to ethylene biosynthesis, ripening and carotenoid biosynthesis at MG, B, O and R fruit developmental stages. *ACS2* and *ACS4*,*ACC synthase 2* and *4*;*ACO1* and *ACO3*,*ACC oxidase 1* and *3*;*RIN*,*RIPENING INHIBITOR; NR, NEVER RIPE; PG, polygalacturonase; DXS1, 1‐deoxy‐D‐xylulose‐5‐phosphate synthase 1; PSY1, phytoene synthase 1; LCY‐B and LCY‐E, B‐* and *E‐lycopene cyclase*. Data are the means of three biological replicates ± SD. *P <* *0.05.

Tomato fruit ripening comprises complex physiological and biochemical processes. To determine the possible effects of *OR* on fruit ripening during late fruit developmental stages, we first measured ethylene production, the indicator of tomato fruit ripening (Giovannoni *et al*., 2017). Ethylene production was not detected at MG stage in all genotypes and reached to peak levels at O stage (Figure 7a). The ethylene levels were significantly boosted at B and O stages in both *AtOR* T1 transgenic fruits in comparison with M82, but remained similar at R stage (Figure 7a). These results showed that *AtOR* did not alter the onset of ripening and could play a role in enhancing ripening.

To see the association of the increased ethylene production with ripening‐related gene expression, we examined the transcript levels of genes related to ethylene biosynthesis (*ACS2*,* ACS4*,* ACO1* and *ACO3*), ripening (*E4*,* RIN*,* NR* and *PG*), and carotenoid biosynthesis (*DXS1*,* PSY1*,* LCY*‐β and *LCY*‐ɛ) by qRT‐PCR. All ethylene biosynthesis related genes examined were dramatically up‐regulated in both *AtOR* ^*WT*^ and *AtOR* ^*His*^ lines particularly at B and O stages (Figure 7b). Similarly, ripening‐related genes of *E4*,* RIN*,* NR* and *PG* were also up‐regulated in *AtOR* lines in comparison with M82 (Figure 7c). With the exception of *DXS1* at MG and O stages and *PSY1* at O stage, the expression of carotenoid biosynthetic genes *PSY1, LcyB* and *LcyE* was generally not greatly affected in the *AtOR* lines (Figure 7d).

### Fruit cellular processes affected by the *AtOR* transgenes

**Figure 8:**
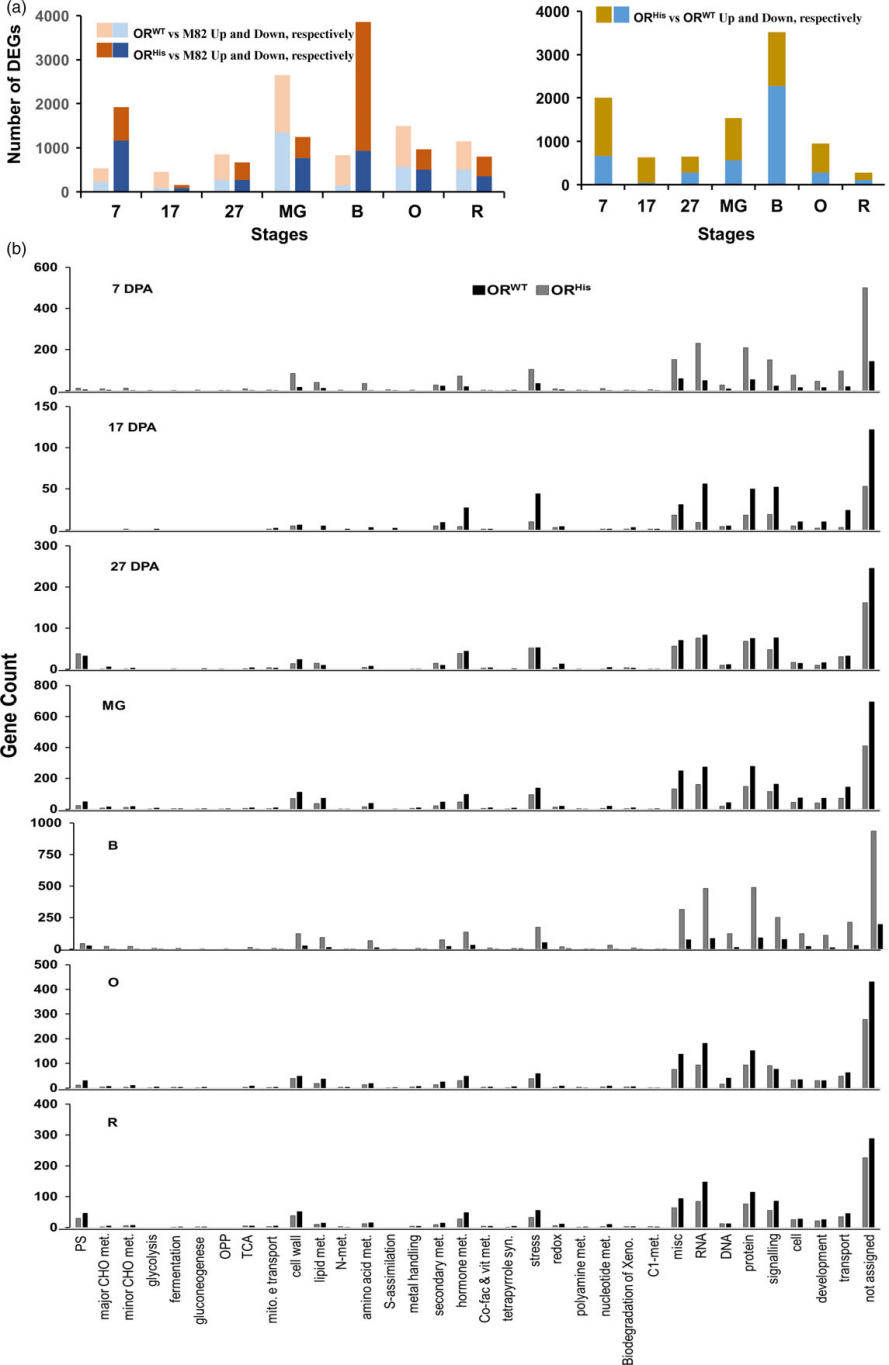
RNA‐seq data analyses. (a) Numbers of DEGs between M82 and *At* *OR*^*WT*^ 20 or *At* *OR*^*H*^ ^*is*^ 21b T1 transgenic lines at each fruit developmental stages (left). Numbers of DEGs between *At* *OR*^*H*^ ^*is*^ 20 and *At* *OR*^*WT*^ 21b lines at each fruit developmental stages (right). Up‐ and down‐regulated genes are also shown. (b) MapMan analysis displays the cellular processes affected by *OR*^*WT*^ and *OR*^*H*^ ^*is*^ at each fruit developmental stages as indicated.

To globally investigate the cellular processes affected by *AtOR* in the transgenic fruit, we compared the transcriptomes of fruit at early (7, 17, and 27 DPA) and late (MG, B, O and R) fruit developmental stages among M82, *AtOR* ^*WT*^ 20 and *AtOR* ^*His*^ 21b lines. A total of 5–13.8 million reads for each of the 63 RNA‐Seq libraries were generated with an average of 83.6% mapped to the tomato genome. A large number of differentially expressed genes (DEGs) between *AtOR* ^*WT*^ or *AtOR* ^*His*^ and M82 at different fruit developmental stages were identified (Figure 8a, Tables S1 and S2). Venn diagram analysis of these DEGs showed many common genes between *AtOR* ^*WT*^ and *AtOR* ^*His*^ lines at all developmental stages (Figure S3a, Table S3). In addition, the analysis also identified 83 and 73 genes that were commonly differentially expressed at 7, 17 and 27 DPA in *AtOR* ^*WT*^ and *AtOR* ^*His*^ lines, respectively, and 105 and 97 genes at last four developmental stages in *AtOR* ^*WT*^ and *AtOR* ^*His*^ lines, respectively, in comparison with M82 (Figure S3b, Table S4).

The DEGs at all developmental stages were functionally categorized using MapMan (Thimm *et al*., 2004). Similar patterns of distribution into functional classes were observed in the *AtOR* ^*WT*^ and *AtOR* ^*His*^ lines (Figure 8b). The overrepresented DEGs in almost all developmental stages belonged to the functional categories for RNA (BIN 27), protein (BIN 29) and signalling (BIN 30), followed by hormone metabolism (BIN 17), stress (BIN 20) and cell wall (BIN 10) (Figure 8b, Table S5).

To investigate the cellular processes affected by both *AtOR* ^*WT*^ and *AtOR* ^*His*^, we also categorized those common DEGs at early three and late four fruit developmental stages in the *AtOR* ^*WT*^ and *AtOR* ^*His*^ lines (Figure S3b, Table S4). The top functional groups were those involved in signalling, protein, RNA and stress at 7, 17 and 27 DPA early fruit developmental stages (Figure S4a) and signalling, protein, RNA and cell wall at MG, B, O and R late ripening stages (Figure S4b). Of particular note, various receptor kinase genes and multiple transcription factors were highly represented in the signalling and RNA groups, respectively (Table S6). Noticeably, while the transcripts of carotenogenic genes in fruit showed under developmental control as reported previously (Ronen *et al*., 1999), the *OR* transgenes in general did not dramatically alter the expression of carotenoid biosynthetic genes during fruit ripening (Figure S5).

To understand the cellular processes specifically affected by *AtOR* ^*His*^ in the transgenic fruit, the DEGs between *AtOR* ^*His*^ and *AtOR* ^*WT*^ were identified (Figure 8a, Table S7). MapMan analysis of DEGs showed that the highly overrepresented groups in almost all developmental stages were RNA, protein and signalling followed by stress (Figure S6). In the RNA functional group, a range from 85% to 97% of DEGs at various developmental stages encoded transcription factors with AP2/EREBP, bHLH, MYB and WRKY families in general being highly represented (Table S8). In the protein group, 74%–87% of DEGs at various stages encoded proteins involved in posttranslational modification and protein degradation (Table S8). Venn diagram analysis of these DEGs uncovered 73 and 17 common genes at early three and late four developmental stages, respectively (Figure S3c). Analysis of the common DEGs by MapMan revealed that the highly represented functional groups were RNA and stress at early fruit developmental stages, and signalling and hormone at late fruit developmental stages (Figure S7, Table S9). The majority of common DEGs at the three early stages were transcription factors of AP2/EREBP, bHLH, MYB and WRKY families in RNA group, and various chaperone proteins in stress group (Table S10). The specifically altered common DEGs at the late fruit developmental stages included many receptor kinases in signalling, and auxin and ethylene genes in hormone group (Table S10). Interestingly, Gag‐Pol polyprotein genes were consistently up‐regulated by *AtOR* ^*His*^ throughout all fruit developmental stages (Table S7).

## Discussion

### Ectopic expression of *AtOR* ^*His*^ further enhances carotenoid levels at all tomato fruit developmental stages

*ORANGE* has been shown as an important genetic tool for carotenoid enhancement in plants (Sun *et al*., 2018; Yuan *et al*., 2015b). Ectopic expression of a wild‐type *OR* leads to carotenoid accumulation in grains of rice and maize (Bai *et al*., 2016; Berman *et al*., 2017), in purple‐fleshed sweetpotato (Park *et al*., 2015) and in calli of sweetpotato and Arabidopsis (Kim *et al*., 2013; Yuan *et al*., 2015a). The ‘golden SNP’ in *OR* is the cause for β‐carotene accumulation in melon fruit (Tzuri *et al*., 2015). Overexpression of a mutated *OR* allele dramatically increases carotenoid levels in transgenic potato tubers and Arabidopsis calli (Campbell *et al*., 2015; Li *et al*., 2012; Lopez *et al*., 2008; Yuan *et al*., 2015a). Here we show that in addition to influencing carotenoid accumulation in nongreen, low carotenoid tissues, *AtOR* ^*His*^ transgene also dramatically enhanced carotenoid levels especially β‐carotene content in tomato flowers and fruit that are already enriched with carotenoids.

Red tomato fruit accumulates substantial amounts of carotenoids, mainly lycopene. Overexpression of carotenoid biosynthetic genes, fibrillin and light signalling genes alters carotenoid levels in tomato fruit (Davuluri *et al*., 2005; Fraser *et al*., 2007; Giliberto *et al*., 2005; McQuinn *et al*., 2017; Nogueira *et al*., 2013; Simkin *et al*., 2007). Up to 1.5‐fold increases of total carotenoid content in transgenic tomato were observed following constitutive expression of *PSY1* (Fraser *et al*., 2007) or *Crt* genes (Nogueira *et al*., 2013). Overexpression of *AtOR* ^*His*^ in tomato fruit conferred a 2.5‐fold increase at R stage (Figure 1e, Table 1), showing the potential of *OR* ^*His*^ for enhancing carotenoid levels in tissues that are already enriched with carotenoids. Moreover, *OR* ^*His*^ promoted an up to 3‐fold increase in β‐carotene, the most potent precursor for provitamin A biosynthesis, in red fruit. The results demonstrate the potential application of *OR* ^*His*^ to further enhance both nutritional and health values in carotenoid‐enriched crops.

Carotenoid accumulation is normally coordinated with the ripening process in tomato fruit. Like the case with *PSY1* (Fraser *et al*., 2007), overexpression of *AtOR* ^*His*^ promoted carotenoid accumulation at early fruit developmental stages, where carotenoid formation proceeds independently from other ripening activities. Phytoene, the carotenoid appeared at B stage of wild‐type fruit, was detected at all fruit developmental stages in the *AtOR* ^*His*^ lines (Table 1). The same profile of carotenoid accumulation in green fruit of the *PSY1* line suggests an increase in PSY activity in the *AtOR* ^*His*^ lines. Indeed, OR is known to post‐transcriptionally regulate PSY enzyme activity (Welsch *et al*., 2018; Zhou *et al*., 2015). In comparison with the *PSY1* line that causes an up to 1.7‐fold increase in total carotenoids in mature green tomato fruit (Fraser *et al*., 2007), a much high fold enrichment (6.4‐fold) was observed at the MG stage in the *AtOR* ^*His*^ lines. Such an increase might be due to the additional role of *OR* ^*His*^ in promoting chromoplast biogenesis with increased sink strength and reduced further metabolism of the synthesized carotenoids (Chayut *et al*., 2017; Yuan *et al*., 2015a).

### Both *AtOR* ^*WT*^ and *AtOR* ^*His*^ regulate plastid size but only *AtOR* ^*His*^ promotes chromoplast development in green fruit

Both *AtOR* ^*WT*^ and *AtOR* ^*His*^ fruit cells contained larger chloroplasts than M82 at early fruit developmental stages (Figure 2). The observed increase in chloroplast sizes suggests a new role of *OR* in regulating plastid expansion. While the mechanism by which OR increases plastid size is not clear, there are a few studies linking genes and processes to the plastid size in tomato fruit. Tomato *high‐pigment* (*hp*) mutants accumulate more carotenoids. *HP‐1* and *HP‐2* encode tomato UV‐damaged DNA‐binding protein 1 (DDB1) and deetiolated1 (DET1) homologs, respectively, in the light signal transduction, and mutations in both genes cause significant increase in chloroplast size in green fruit (Cookson *et al*., 2003; Kolotilin *et al*., 2007; Liu *et al*., 2004). *HP‐3* encodes ZEP in the carotenoid biosynthetic pathway. Its mutation leads to 30% larger plastid size in green fruit (Galpaz *et al*., 2008). In these *HP* mutants, the larger plastid sizes are accompanied with increased plastid numbers. However, the enlarged plastid size in these *AtOR* lines appeared not to link with increased plastid number, indicating a different regulation of plastid size by *OR* from the *HP* genes. OR protein shares structural similarity with maize Bundle Sheath Defective 2 (BSD2) (Lu *et al*., 2006). A recent study reveals that BSD2 also mediates chloroplast size in maize bundle sheath cells by unknown mechanisms (Salesse‐Smith *et al*., 2017).

The mutated *OR* alleles are known to initiate chromoplast formation from proplastids and/or leucoplasts (Lu *et al*., 2006; Yuan *et al*., 2015a). A study with the melon *lowβ* mutant, an *OR* gene non‐sense mutation, suggests that *OR* ^*His*^ is also involved in chloroplast‐to‐chromoplast transition during melon fruit development (Chayut *et al*., 2017). Here we show that *AtOR* ^*His*^ promoted chromoplast differentiation likely from proplastids at very early stages of fruit development (Figure 2). Fully developed chromoplasts were seen at 7 DPA, and chromoplasts with red crystal structures were observed at 27 DPA in the *AtOR* ^*His*^ lines. In addition, intermediate plastids containing both carotenoids and chlorophylls were observed at early fruit development stages, showing chromoplast biogenesis from chloroplasts (Egea *et al*., 2011). The presence of intermediate plastids indicates the involvement of *AtOR* ^*His*^ in chloroplast‐to‐chromoplast transition, perhaps resulting from its roles in regulating chromoplast development and carotenoid accumulation. A mechanism of metabolite‐induced plastid transition was proposed following the observation of chromoplast‐like structures at early fruit developmental stages in the *PSY‐1* transgenic tomato (Fraser *et al*., 2007). Investigation of the basis of β‐carotene accumulation in fruit of melon *lowβ* mutant also suggests that OR^His^ has a metabolic arrest function to confer chromoplast formation (Chayut *et al*., 2017).

### Novel roles of *AtOR* in promoting early flowering, fruit set and seed production

One clear phenotype in the reproductive tissues of the *AtOR* transgenic lines was early flowering. This is particularly interesting given the importance of flowering initiation for fruit production. *AtOR* was found to alter the expression of a number of genes involved in flowering in tomato. *SP* and *TMF* are negative regulators of flowering and *SFT* and *SlZFP2* are positive regulators of flowering in tomato (Lifschitz and Eshed, 2006; MacAlister *et al*., 2012; Pnueli *et al*., 1998; Weng *et al*., 2016). Analysis of their expression in shoot meristems, flowers and young fruit at 2–3 DPA revealed that *AtOR* down‐regulated *SP* and *TMF* and up‐regulated *SFT* and *SlZFP2* (Figure 4), suggesting the potential roles of *OR* in mediating flowering time via affecting some flowering gene expression in tomato.

Fruit set is a crucial stage in fruit production and represents an agronomically important trait. It begins with pollination and subsequent conversion of the ovary into a developing fruit. Here we found that the fruit numbers in 10‐ and 15‐week‐old plants were significantly increased in both *AtOR* ^*WT*^ and *AtOR* ^*His*^ transgenic lines. Furthermore, fruit seed number also showed a significant increase (Figure 5). A number of factors and genes are known to affect fruit set and seed development in tomato (Ruan *et al*., 2012). Fruit set is limited by carbon resources and the capacity of very young fruit to utilize them (D'Aoust *et al*., 1999). Cell wall invertase LIN5 and its inhibitor INH1 that control fruit hexose levels have been shown to play a major role for fruit and seed set in tomato (Palmer *et al*., 2015; Zanor *et al*., 2009). Suppression of *LIN5* expression leads to fruit and seed abortion in tomato (Zanor *et al*., 2009). The transcript level of *LIN5* is induced during ovary‐to‐fruit transition (Palmer *et al*., 2015). Increase in the invertase activity by silencing *INHI* promotes fruit set under heat stress (Liu *et al*., 2016). Examination of some sucrose hydrolysis gene expression revealed that the young fruitlets of both *AtOR* overexpression lines contained significantly higher *LIN5* transcript and lower *INH1* expression than M82 (Figure 6) to correlate with enhanced fruit set and seed production. The demonstration of *OR* in affecting fruit set adds OR as a potential new genetic element in influencing fruit development.

### *AtOR* primarily affects cellular processes related to RNA, protein and signalling in tomato fruit

The functional roles of *OR* in plants remain to be fully elucidated. Global transcriptome analysis revealed that genes involved in the cellular processes of RNA, protein and signalling were clearly enriched in fruit of *AtOR* ^*WT*^ and *AtOR* ^*His*^ lines. These genes included a large number of transcription factors particularly *AP2/EREBP*,* bHLH*,* MYB* and *WRKY* family genes, protein posttranslational modification and ubiquitin directed degradation genes, and receptor kinases and calcium signalling genes (Table S5). Some of them are known to influence diverse metabolism and processes in tomato (Giovannoni *et al*., 2017; Seymour *et al*., 2013). While transcript changes do not necessary match activities, the altered expression of genes in these functional groups provided hints for further elucidation of the bases underlying the *OR*‐mediated carotenogenesis, plastid development, flowering and fruit and seed set in tomato fruit. For example, FtsH metalloproteases affect chloroplast size and number oppositely in Arabidopsis (Kadirjan‐Kalbach *et al*., 2012). A tomato *FtsH* homolog (*Solyc02 g062550*) was observed to be differentially regulated by both *OR* ^*WT*^ and *OR* ^*His*^ during early stages of fruit development (Table S9), suggesting a potential role of *OR* in regulating *FtsH* to mediate plastid size.

While both *AtOR* ^*WT*^ and *AtOR* ^*His*^ influenced plastid development and early fruit development, *AtOR* ^*His*^ specifically influenced chloroplast‐to‐chromoplast conversion and induced carotenoid accumulation at early fruit developmental stages. The genes related to the cellular processes of RNA and stress at the three early fruit developmental stages were overrepresented in *AtOR* ^*His*^ in comparison with *AtOR* ^*WT*^ lines. Previous transcriptome analysis of orange vs green flesh melon fruit also identified RNA and stress as the enriched processes (Chayut *et al*., 2015). All the common genes in the RNA bin are transcription factors (Table S10). Among the 18 TFs included 4 bHLH, 4 AP2/EREBP and 4 WRKY. The bHLH TFs such as PIFs regulate carotenoid biosynthesis and plastid transition (Llorente *et al*., 2016). A bHLH TF was recently shown to negatively regulate chlorophyll and carotenoid genes in affecting fruit pigment in tomato (Zhu *et al*., 2017). AP2/EREBPs have been implicated in various hormones‐related signalling pathways including affecting ethylene to impact carotenoid production (Liu *et al*., 2015). They are also the highly represented TFs that are differentially expressed in melon fruit (Chayut *et al*., 2015). Recent work indicates that both AP2/EREBPs and WRKYs play important roles in plastid retrograde signalling (Phukan *et al*., 2016, 2017). Heat shock proteins are associated with chromoplast differentiation and carotenoid accumulation in tomato (D'Andrea *et al*., 2018; Neta‐Sharir *et al*., 2005). Among the 13 common genes at *AtOR* ^*His*^ early fruit developmental stages, 8 of them are heat shock proteins (Table S9). The high abundance of TFs and heat shock proteins suggests roles of them in the *AtOR* ^*His*^ mediated chloroplast‐to‐chromoplast transition and carotenoid accumulation, which are to be explored.

In conclusion, this study provides new information for the novel roles of *OR* in mediating carotenogenesis, plastid development and fruit development. In addition to regulating carotenoid accumulation in low carotenoid containing tissues, *OR* ^*Hi*s^ can also greatly enhanced carotenoid levels in tissues that are already enriched with carotenoids. Moreover, *OR* was found to promote early flowering and fruit set, two agronomically important traits for fruit production, along with increased seed production. The early flowering and fruit set have commercial potentials which include the potential of increasing fruit yield, early marketing in season, and avoiding of the chilly injury in cold areas. The increased seed production in fruit could meet large‐scale commercial planting. Study here provides evidence for a broad application of *OR* in engineering crops not only for nutritional quality improvement, but also for fruit production.

## Experimental procedures

### Plant materials and *Agrobacterium*‐mediated transformation

Tomato (*Solanum lycopersicum*) cv M82 was used in this study. The *AtOR* ^*WT*^ and *AtOR* ^*His*^ overexpression constructs (Yuan *et al*., 2015a) were transformed into the tomato cotyledon explants according to Gupta and Van Eck (2016). The individual transgenic lines and nontransformed M82 plants were grown in a greenhouse at 26 °C with 12 h light in day and at 20 °C for 12 h in evening until maturity. The T1 fruit was tagged at size of 1 cm, which was corresponded to 7 DPA as determined here and described previously (Alba *et al*., 2005). Fruit were harvested at 7, 17, 27, 38 (mature green, MG), 43 (breaker, B), 46 (orange, O) and 50 (red, R) DPA stages. Outer pericarp of fruit (5–10) was pooled at each developmental stage, frozen in N2 and stored at −80 °C until use.

### Protein extraction and Western blot analysis

Total protein extraction from tomato leaf and fruit tissues and Western blot analysis were performed as described previously (Yuan *et al*., 2015a). The total proteins were separated by 10% SDS‐PAGE gels and blotted onto nitrocellulose membranes. The membranes were then blocked and incubated with primary anti‐OR antibody (Lu *et al*., 2006), anti‐PSY antibody (Yuan *et al*., 2015a) or plant anti‐actin antibody (Sigma Aldrich, St. Louis, MO, USA). Signals were detected by ECL Prime Western Blotting Detection Reagent (GE Healthcare, Menlo, CA, USA).

### Confocal microscopy

To measure the autofluorescence of tomato plastids, three 7, 17 and 17 DPA fruit of M82, *At*OR^WT^ and *At*OR^His^ lines were harvested. A piece of equatorial pericarp was cut into thin slices. The autofluorescences of chlorophylls and carotenoids were monitored under Leica TCS SP5 Laser Scanning Confocal Microscope, using the setting described by D'Andrea *et al*. (2014). The 488 nm light was used as excitation source. Chlorophyll fluorescence was taken at 650–700 nm, and carotenoid fluorescence was taken at 500–550 nm.

To measure plastid sizes, plastid pictures in cells of 7 DPA fruit from M82, *AtOR* ^*WT*^ 20 and *AtOR* ^*His*^ 23a T1 transgenic plants were taken under Confocal Microscope with the settings as above. The diameters of plastids were measured by the scale tool of the Leica Application Suite software.

### Carotenoid extraction and analysis

Carotenoids from tomato fruit pericarp (400 mg) were extracted as described by Lopez *et al*. (2008). Carotenoids from flowers (100 mg) were extracted and saponified according to the method used (Zhang *et al*., 2014). Carotenoid analysis was carried out using Acquity UPC^2^ ™ HSS C_18_ SB 1.8 μm column (3.0 × 100 mm) in a Waters UPC^2^ system in a total of 6 min. The initial concentrations for CO_2_ and methanol in UPC^2^ system were 97.5% and 2.5%, respectively, for 2 min followed by a gradient to 75% CO_2_ and 25% methanol in 2 min and back to initial condition in 2 min. All samples were analysed with three biological replicates.

### Fruit set analysis

Fruit in five trusses from 10‐ and 15‐week‐old plants for each line were counted and averaged. The total number of fruit for each line was also determined in 10‐ and 15‐week‐old plants. Seed numbers were counted from 15 to 20 fruit at R stage with approximately the same size.

### Ethylene and sugar assay

Fruit from MG, B, O and R stages (5–6 fruit for each stage) of M82, *AtOR* ^*WT*^ 20 and *AtOR* ^*His*^ 21b T1 transgenic lines was sealed for 2 h in glass jars to collect ethylene (Barry *et al*., 2012). A 1‐mL of headspace sample was analysed by gas chromatograph (Carle AGC series 100) equipped with an activated alumina column and flame ionization detector. Sugar content of fruit pericarp (100 mg) was measured using phenol‐sulphuric acid method (Masuko *et al*., 2005).

### Subcellular fractionation of chromoplasts

Subcellular fractionation of chromoplasts was carried out as described by Nogueira *et al*. (2013). Tomato fruit at the stage of B + 3 to 5 days from M82, *AtOR* ^*WT*^ 20 and *AtOR* ^*His*^ 21b T1 transgenic plants were harvested. Chromoplasts from pericarps (100 g) were isolated, broken by a handheld potter homogenizer and fractionated by ultracentrifugation at 100 000 g for 17 h at 4 °C in a discontinuous sucrose gradient consisting of 6 mL of 38%, 6 mL of 20%, 4 mL of 15% and 8 mL of 5% sucrose buffer. One mL fractions were collected from top of the gradients and used for carotenoid analysis by UPC^2^ and Western blot analysis following extraction of proteins from the fractions according to Nogueira *et al*. (2013).

### RNA extraction and qRT‐PCR

Total RNA from tomato leaf, flower or meristem tissues (100 mg) was extracted using Trizol reagent (Life Technologies, Germantown, MD, USA). Fruit total RNA was extracted using RNeasy Plant Mini Kit according to manufacturer's manual (Qiagen, Carlsbad, CA, USA). Real‐time RT‐PCR was carried out using gene‐specific primers (Table S11) and SYBR Green Supermix in ABI 7500 Real‐Time PCR system (Zhou *et al*., 2011). Tomato *Actin* gene was used as an internal control and relative changes in expression levels were analysed using 2^−ΔΔCT^ method (Lyi *et al*., 2007). The experiments were carried out with at least two technical trials for three biological replicates.

### Construction of RNA‐Seq libraries and data analysis

Total RNA from fruit of M82, *AtOR* ^*WT*^ 20 and *AtOR* ^*His*^ 21b T1 transgenic lines at 7, 17, 27 DPA, MG, B, O and R developmental stages were used. Strand‐specific RNA‐Seq libraries were prepared following the method as described (Zhong *et al*., 2011). PolyA RNA from total RNA was isolated by oligodT beads, fragmented and used for first‐strand cDNA synthesis. The second‐strand cDNA was synthesized using a dUTP mix followed by end‐repair, dA‐tailing and adapter ligation. Sixty‐three single barcoded RNA‐Seq libraries (seven stages × three genotypes × three replicates) were sequenced on three lanes of an Illumina HiSeq 2500 in the Genomics Facilities at the Institute of Biotechnology at Cornell University (www.biotech.cornell.edu/brc/genomics-facility).

Raw RNA‐seq reads were processed using Trimmomatic (Bolger *et al*., 2014) to remove adaptors and low‐quality sequences. Reads shorter than 40 bp were discarded. RNA‐Seq reads were then aligned to the ribosomal RNA database (Quast *et al*., 2013) using Bowtie (Langmead *et al*., 2009) and the mappable reads were discarded. The resulting high‐quality cleaned reads were aligned to the tomato Heinz genome (Tomato Genome Consortium, 2012) using HISAT (Kim *et al*., 2015). Following alignments, raw counts for each tomato gene were derived and normalized to reads per kilobase of exon model per million mapped reads (RPKM). Differentially expressed genes (DEGs) were identified with the edgeR package (Robinson *et al*., 2010) using the cut‐off criteria of an expression fold change >3 and adjusted *P* value <0.01

MapMan software was used for identifying the functional groups of the DEGs (Thimm *et al*., 2004). Venn diagrams were prepared using Bioinformatics & Evolutionary Genomics software (http://bioinformatics.psb.ugent.be/webtools/Venn/). The raw sequencing data were deposited to NCBI under the accession number SRP126696.

## Conflict of interest

The authors declare that they have no conflict of interests.

## Author contributions

MY, ZS and HY performed the experiments. MY, SZ, QM and ZF carried out or supervised transcriptome data analysis. TWT helped and guided carotenoid analysis by UPC^2^. JV aided with ethylene analysis. YX constructed RNA‐seq libraries. JVE supervised tomato transformation. ST, YT and JJG provided suggestions on the research design and assisted data analysis and interpretation. MY and LL wrote the manuscript. All authors contributed to the final manuscript.

## Supporting information


**Figure S1** Western blot analysis of OR protein in leaves of *AtOR* ^*WT*^ and *AtOR* ^*His*^ T0 transgenic tomato lines.
**Figure S2** OR expression and carotenoid levels in leaves of M82 and the *AtOR* T1 transgenic plants.
**Figure S3** Venn diagram analyses of RNA‐Seq data.
**Figure S4** MapMan analysis of the common DEGs at 3 early and 4 late fruit developmental stages of OR^WT^ vs M82 and OR^His^ vs M82.
**Figure S5** Expression of carotenoid metabolic pathway genes during tomato fruit ripening in M82, *AtOR* ^*WT*^ and *AtOR* ^*His*^ lines.
**Figure S6** MapMan analysis of the DEGs between *AtOR* ^*His*^ 20 and *AtOR* ^*WT*^ 21b lines at all fruit developmental stages.
**Figure S7** MapMan analysis of the common DEGs at 3 early and 4 late fruit developmental stages.

**Table S1** DEGs of *AtOR* ^*WT*^ vs M82 at all fruit developmental stages.

**Table S2** DEGs of *AtOR* ^*His*^ vs M82 at all fruit developmental stages.

**Table S3** Common DEGs between *AtOR* ^*WT*^ vs M82 and *AtOR* ^*His*^ vs M82 at all fruit developmental stages.

**Table S4** Common DEGs of *AtOR* ^*WT*^ or *AtOR* ^*His*^ among early (7–27 DPA) or late (MG‐R) fruit developmental stages.

**Table S5** MapMan groups of DEGs of *AtOR* ^*WT*^ vs M82 and *AtOR* ^*His*^ vs M82 at all fruit developmental stages.

**Table S6** MapMan groups of common DEGs of *AtOR* ^*WT*^ vs M82 and *AtOR* ^*His*^ vs M82 among early (7–27 DPA) and late (MG‐R) fruit developmental stages.

**Table S7** DEGs between *AtOR* ^*WT*^ and *AtOR* ^*His*^ lines at all fruit developmental stages.

**Table S8** MapMan groups of DEGs between *AtOR* ^*His*^ and *AtOR* ^*WT*^ at all fruit developmental stages.

**Table S9** Common DEGs between *AtOR* ^*His*^ and *AtOR* ^*WT*^ among early (7–27 DPA) and late (MG‐R) fruit developmental stages.

**Table S10** MapMan groups of common DEGs between *AtOR* ^*His*^ and *AtOR* ^*WTs*^ among early (7‐27 DPA) and late (MG‐R) fruit developmental stages.

**Table S11** List of primers used in this study.
