# Supplementary material for: Ectopic expression of ORANGE promotes carotenoid accumulation and fruit development in tomato
Source: Plant Biotechnol J. 2018 May 31;17(1):33–49. doi: 10.1111/pbi.12945 (PMC6330546; doi:10.1111/pbi.12945)
Supplement: Supplementary file 1 — Figure S1 Western blot analysis of OR protein in leaves of AtOR WT and AtOR His T0 transgenic tomato lines. Figure S2 OR expression and carotenoid levels in leaves of M82 and the AtOR T1 transgenic plants. Figure S3 Venn diagram analyses of RNA‐Seq data. Figure S4 MapMan analysis of the common DEGs at 3 early and 4 late fruit developmental stages of ORWT vs M82 and ORHis vs M82. Figure S5 Expression of carotenoid metabolic pathway genes during tomato fruit ripening in M82, AtOR WT and AtOR His lines. Figure S6 MapMan analysis of the DEGs between AtOR His 20 and AtOR WT 21b lines at all fruit developmental stages. Figure S7 MapMan analysis of the common DEGs at 3 early and 4 late fruit developmental stages. [file PBI-17-33-s006.pdf]

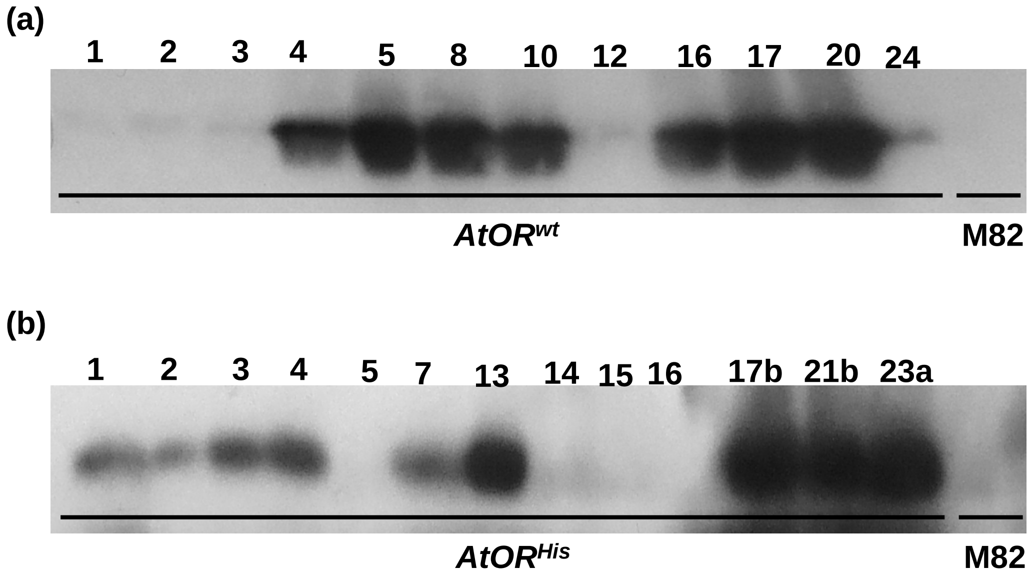

Figure S1: Western-blot analysis of OR protein levels in leaves of *AtOR*^{wt} (a) and *AtOR*^{His} (b) T0 transgenic tomato lines. A total of 20 μg proteins were loaded for each sample. The numbers above gels indicate independent transgenic lines.

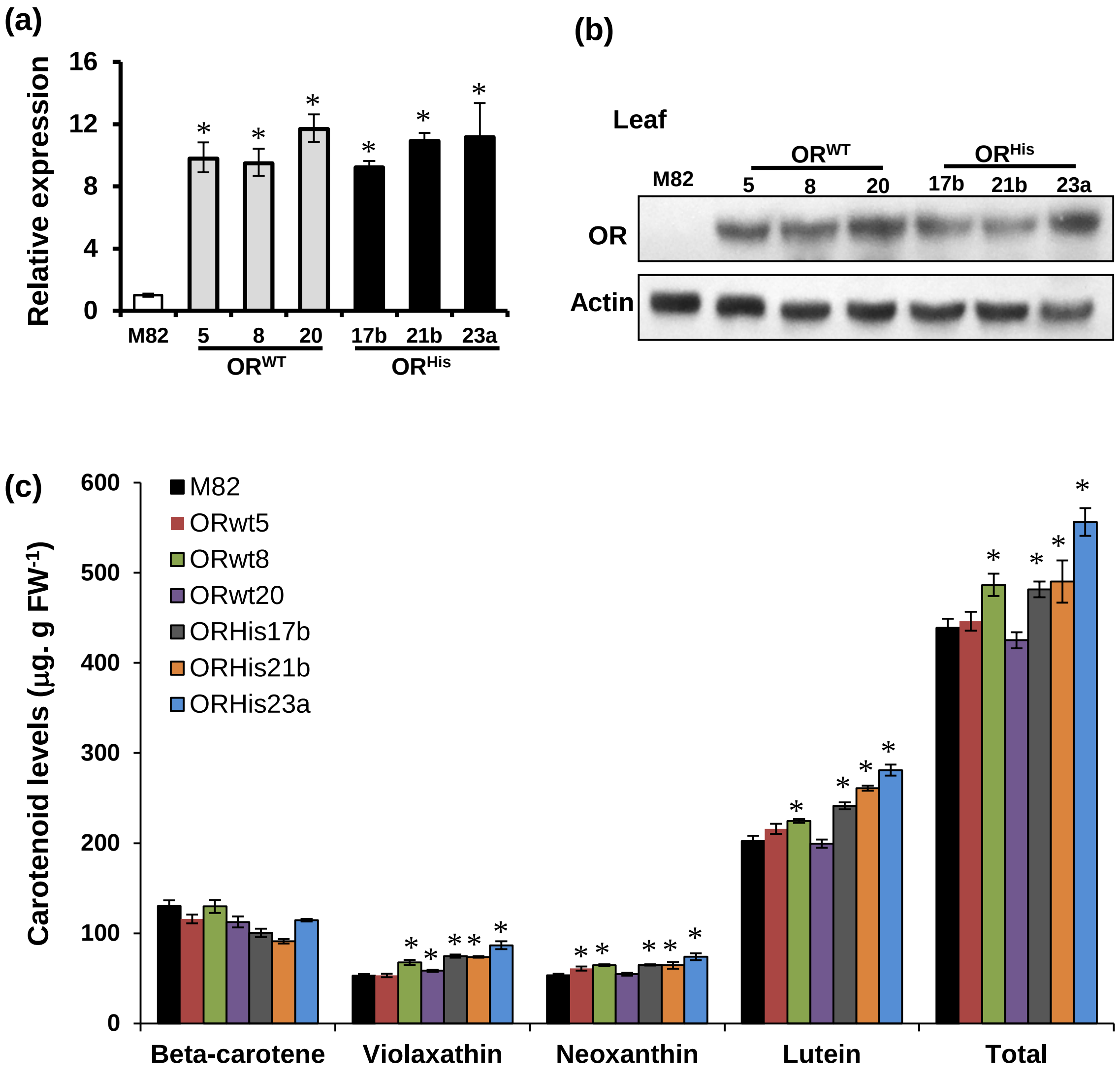

Figure S2 OR expression and carotenoid levels in leaves of M82 and the *AtOR* T1 transgenic plants. (a) qRT-PCR analysis of the *OR* gene expression in leaves of 4-week old plants. (b) Western-blot analysis of the OR protein levels in leaves of 4-week old plants. Actin shows protein loading. (c) Total carotenoid levels and composition in leaves of 4-week old plants. Data are the means of three biological replicates ± SD. * $P < 0.05$. FW, fresh weight

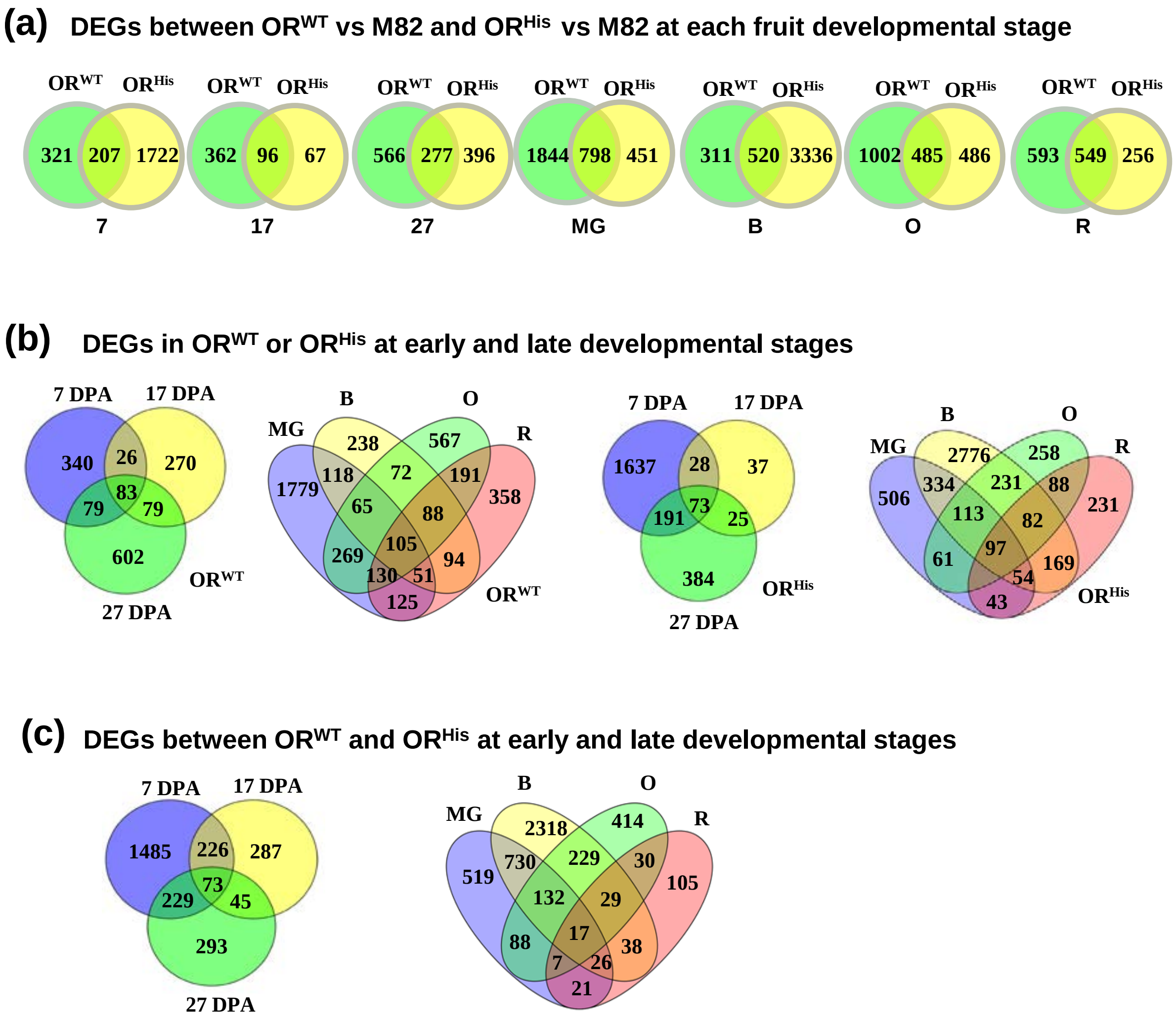

Figure S3 Venn diagram analyses of RNA-Seq data. (a) Venn diagrams showing the number of unique and common DEGs between *AtOR*^{WT} vs M82 and *AtOR*^{His} vs M82 at various fruit developmental stages. (b) Venn diagrams showing the number of unique and common DEGs for *AtOR*^{WT} and *AtOR*^{His} lines at early (7, 17, 27 DPA) and late (MG, B, O, R) fruit developmental stages. (c) Venn diagrams showing the number of unique and common DEGs between *AtOR*^{WT} and *AtOR*^{His} lines at early (7, 17, 27 DPA) and late (MG, B, O, R) fruit developmental stages. The expression ratio of >3 and adjusted P < 0.01were used for the analyses. DPA, days post-anthesis; MG, mature green; B, breaker; O, orange; R, red

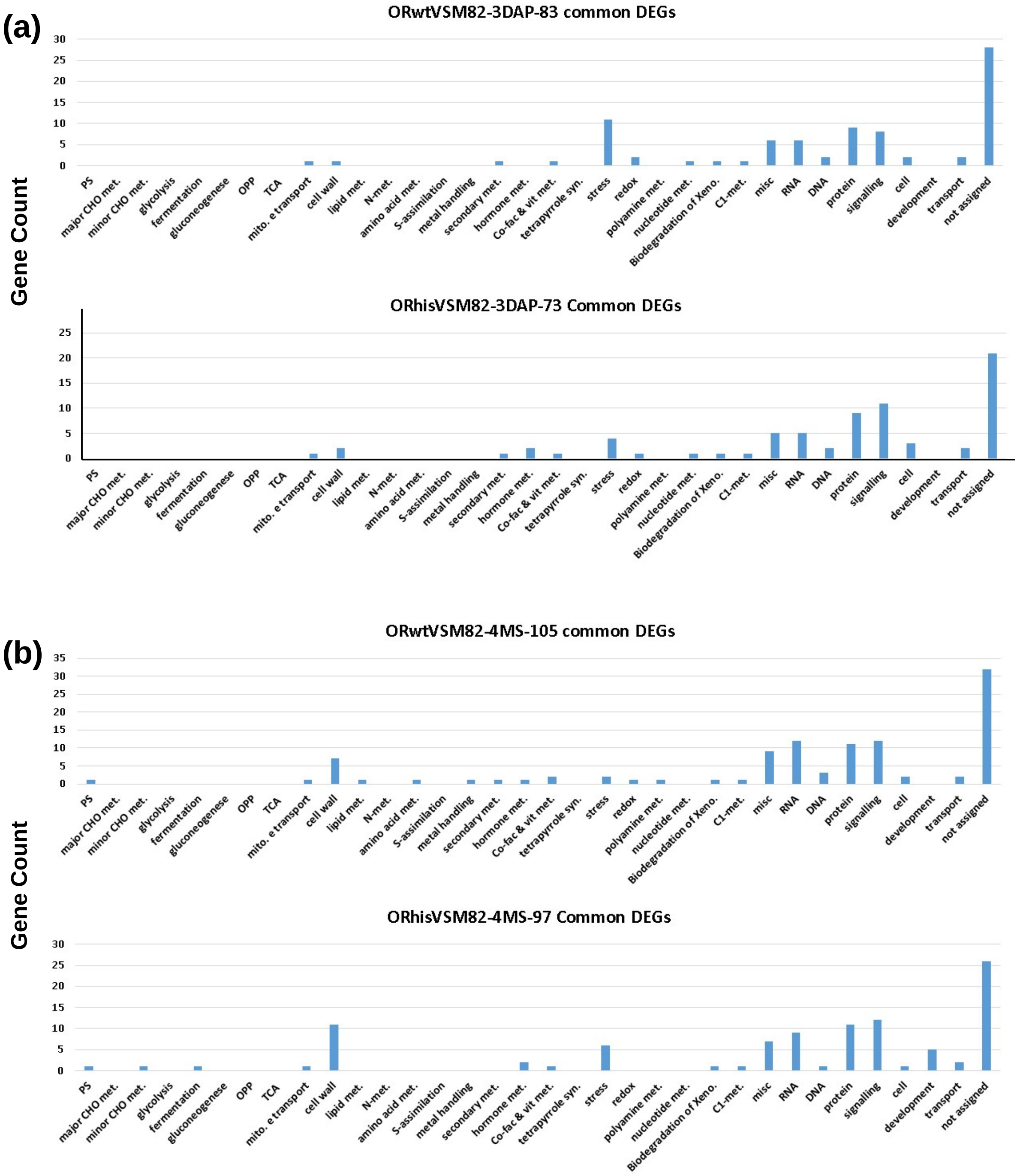

Figure S4 MapMan analysis of the common DEGs at 3 early (A) and 4 late (B) fruit developmental stages of OR^{WT} vs M82 and OR^{His} vs M82. 3DAP, three early stages at 7, 17, and 27 days post-anthesis; 4MS, four mature stages at mature green, breakers, orange, and red

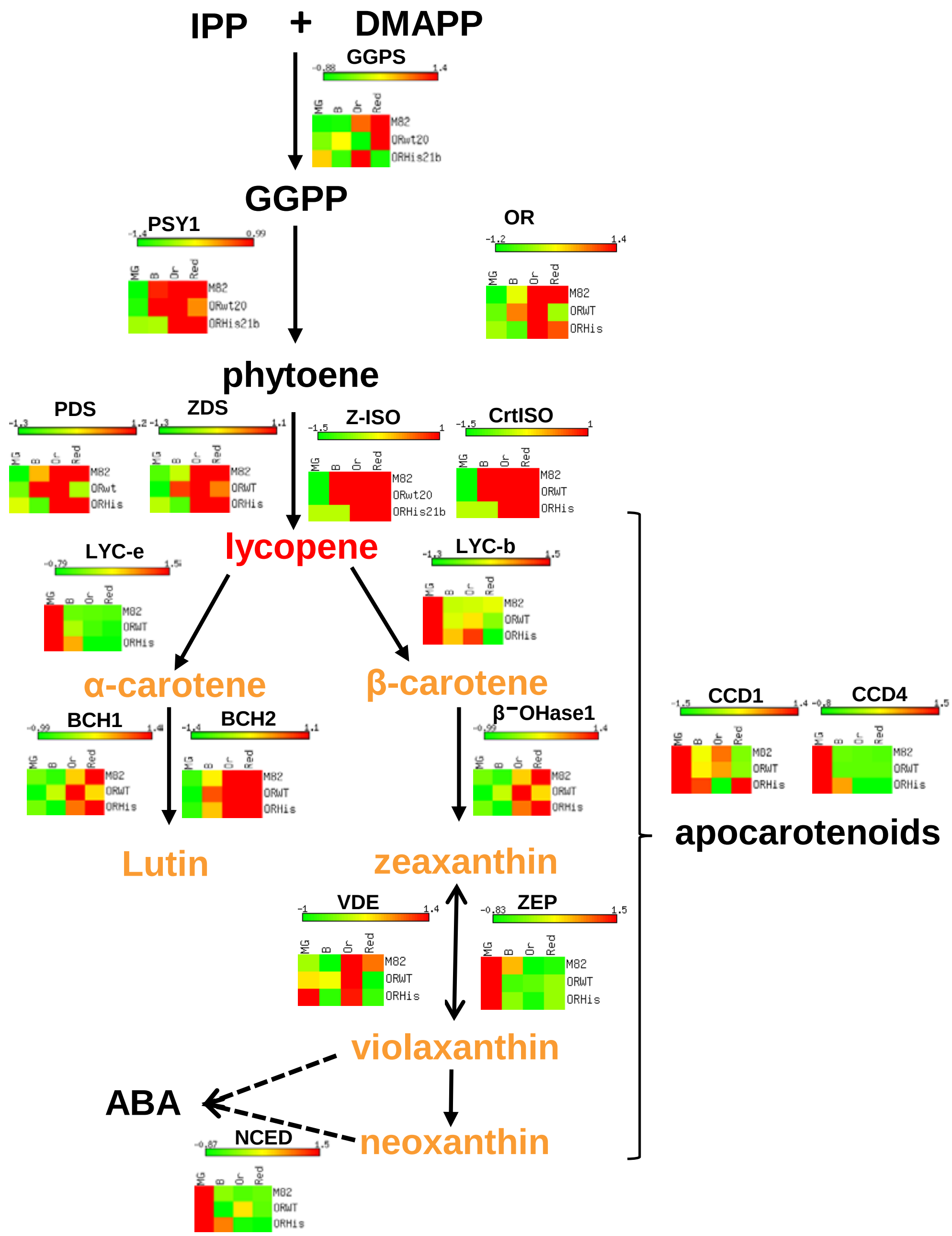

Figure S5 Expression of carotenoid metabolic pathway genes during tomato fruit ripening in M82, *AtOR*^{WT} and *AtOR*^{His} lines. Heatmaps show the average RPKM of three biological repeats at each fruit developmental stage. The gene IDs are GGPS (Solyc09g008920); PSY1 (Solyc03g031860); OR (Solyc03g093830); DXS(Solyc01g067890); DXR (Solyc03g114340); PDS (Solyc03g123760); ZDS (Solyc01g097810); CrtISO (Solyc12g098710); LYC-e (Solyc12g008980); LYC-b (Solyc04g040190); BCH1 (Solyc06g036260); BCH2 (Solyc03g007960); β-OHase1 (Solyc03g007960); VDE (Solyc04g050930); ZEP (Solyc02g090890); NCED (Solyc08g016720); CCD1 (Solyc01g087250); and CCD4 (Solyc08g075490).

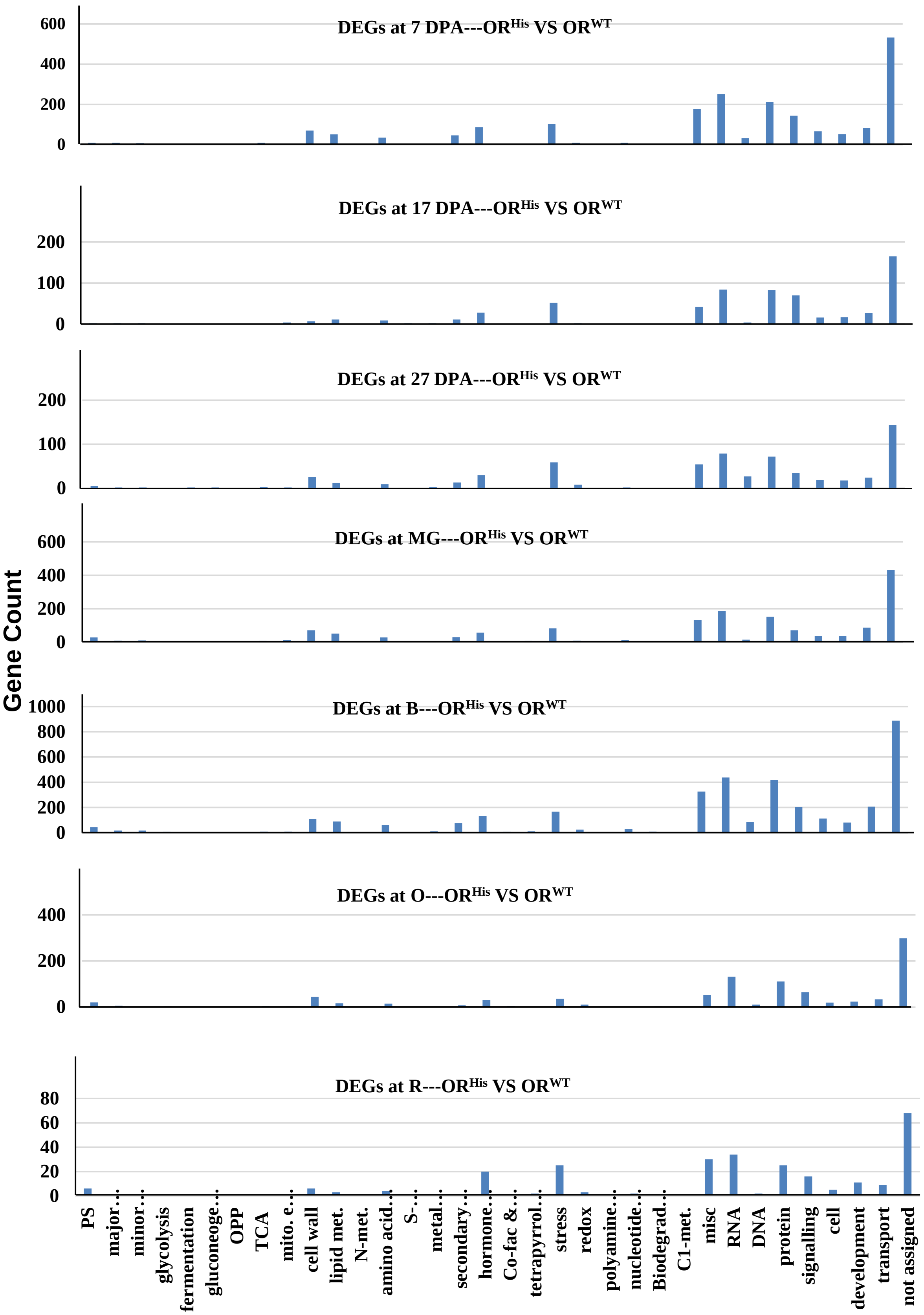

Figure S6 MapMan analysis of the DEGs between *AtOR*^{His} 20 and *AtOR*^{WT} 21b lines at each fruit developmental stages. DPA, days post-anthesis; MG, mature green; B, breakers; O, orange; R, red stage

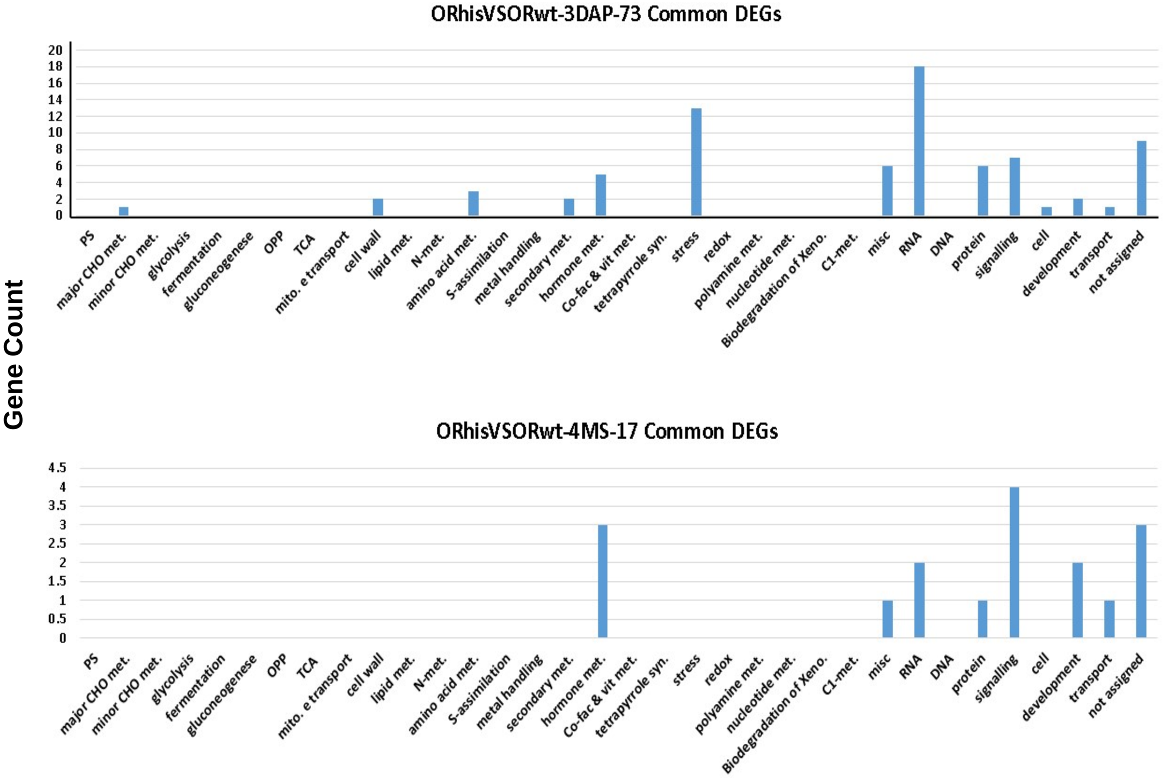

Figure S7 MapMan analysis of the common DEGs at 3 early (A) and 4 late (B) fruit developmental stages between *AtOR*^{His} 20 and *AtOR*^{WT} 21b lines . 3DAP, three early stages at 7, 17, and 27 days post-anthesis; 4MS, four mature stages at mature green, breakers, orange, and red
