# Supplementary material for: Ectopic expression of ORANGE promotes carotenoid accumulation and fruit development in tomato
Source: Plant Biotechnol J. 2018 May 31;17(1):33–49. doi: 10.1111/pbi.12945 (PMC6330546; doi:10.1111/pbi.12945)
Supplement: Supplementary file 12 — Table S11 List of primers used in this study. [file PBI-17-33-s008.pdf]

Table S11. Primers used in this study

Primers used in this study for qRT-PCR			
AtOR-F	CGTAAAGCAGCAAGAGCACA	*AtOR*-R	GTTTTGGGCGGTGATAGAGA
SlOR-F	GAGTGCTGAGCTTGGGATAATA	*SlOR*-R	CCGGCAATGAGGGATATACAA
SlLIN5-F	GATCCTAGCAGAAATCGAAGGG	*SlLIN5*-R	GCCATACTTGTCGCGGAATA
SlLIN6-F	GTAAGTGGATCAAGCCCGATAA	*SlLIN6*-R	CTCACACTCCCAACCAATACTC
SlSS1-F	CTGTCCGTCCCTGAGTATTTG	*SlSS1*-R	TTGGTGAGGGTTGGTTTAGG
SlSS3-F	CTTGCTGTTTAGCGATGTTGAG	*SlSS3*-R	GTTCTTCACTCGGTCCAATCT
SlVI-F	CGCCTCTCGTTACACATTACTC	*SlVI*-R	TCGGAAAGAAGGCTACAGAAAG
SlINH1-F	GCATTCTAATCCTCCTCAAGC	*SlINH1*-R	CTTCACATTCTTGTGCATCACC
SlDXS1-F	TGACCATGGATCTCCTGTTG	*SlDXS1*-R	GCCTCTCTGGTTTGTCCAAG
SlPSY1-F	GCCATTGTTGAAAGAGAGGGTG	*SlPSY1*-R	AGGCAAACCAACTTTTCCTCAC
SlLCY-E-F	GCCACAAGAACGAAAACGAC	*SlLCY-E*-R	CGCGGAAAAATGACCTTATC
SlLCY-B-F	TTGTGGCCCATAGAAAGGAG	*SlLCY-B*-R	GGCATCGAAAAACCTTCTTG
SlACS2-F	CGTTTGAATGTCAAGAGCCAGG	*SlACS2*-R	TCGCGAGCGCAATATCAAC
SlACS4-F	CACACCCTATTACCCAGGATTT	*SlACS4*-R	GGCCTCTTCGATAGCTTCTATT
SlACO3-F	GAATCCTCCCAGTCTACAAGAAT	*SlACO3*-R	GACCGAGACCTTCAGACAATAA
SlACO1-F	GCGCCATCTTCCTACTTCTAAT	*SlACO1*-R	TAACTCCTCAGCCAATTTCTCC
SlNR-F	CTCCCAGAGGCAGATTGAAC	*SlNR*-R	TTCACAGACATCCCACCATC
SlRIN-F	GCTAGGTGAGGATTTGGGACAA	*SlRIN*-R	AATTTGCCTCAATGATGAATCCA
SlPG-F	GTGGTCCAGGTCATGGTATAAG	*SlPG*-R	CCTGCCAAGTCTTGATCCTAA
SlE4-F	TAGCAAGTCAACCACCAATCC	*SlE4*-R	ACCCTCTGGAAAGCCAATTC
SlSP-F	CCAGATGTTCTTGGTCCTAGTG	*SlSP*-R	CATACCCAACCACTTCTCTTCC
SlSFT-F	TGTTGTTGGTCGTGTGGTAG	*SlSFT*-R	GCCTAAGCTCGCATCCATTA
SlTMF-F	TCCTGGACCTAGTGATCCTTAT	*SlTMF*-R	GGTCTCATAGCTCACCAATTCT
SlZFP2-F	GAACCAAACACGGCCTTAAAC	*SlZFP2*-R	GGCTCAACCGGGCTTAAA
SlActin-F	CCTTCCACATGCCATTCTCC	*SlActin*-R	CCACGCTCGGTCAGGATCT
